# Physicochemical and functional characterization of MYL-1501D, a proposed biosimilar to insulin glargine

**DOI:** 10.1371/journal.pone.0253168

**Published:** 2021-06-16

**Authors:** Parag Goyal, Harish Venkatraman Pai, Phanichand Kodali, Bhavesh Vats, Navratna Vajpai, Shankara Annegowda, Krishnappa Mane, Shamini Mohan, Shruti Saxena, Anil Bangalore Veerabhadraia, Milee Palande, Preethy Sasankan Nair, Digvijay Chandrashekar More, Umamaheshwara Rao Karudumpa, Kunala Jyothirmai, Adroha Bhattacharya, Frida Almeida, Santosh Gulab Khyade, Shankara Gouda, Daniel J. Ranayhossaini, Praveen Reddy Moole, Jeffrey P. Smith, Abhijit Barve, Ramakrishnan Melarkode, Rajesh Ullanat

**Affiliations:** 1 Viatris Inc, Canonsburg, PA, United States of America; 2 Biocon Research Limited, Bangalore, India; 3 Viatris Inc, Hyderabad, India; Cairo University, EGYPT

## Abstract

Insulin glargine is a long-acting analogue of human insulin that has been used to manage hyperglycemia in patients with diabetes mellitus (DM) for nearly 20 years. Insulin glargine has a relatively constant concentration-time profile that mimics basal levels of insulin and allows for once-daily administration. MYL-1501D is a biosimilar insulin glargine designed to offer greater access of insulin glargine to patients, with comparable efficacy and safety to the marketed reference product. We conducted a comprehensive panel of studies based on a formal analysis of critical quality attributes to characterize the structural and functional properties of MYL-1501D and reference insulin glargine products available in the United States and European Union. MYL-1501D was comprehensively shown to have high similarity to the reference products in terms of protein structure, metabolic activity (both *in vitro* cell-based assays and *in vivo* rabbit bioassays), and *in vitro* cell-based assays for mitogenic activity. The structural analyses demonstrated that the primary protein sequence was identical, and secondary and tertiary structures are similar between the proposed biosimilar and the reference products. Insulin receptor binding affinity and phosphorylation studies also established analytical similarity. MYL-1501D demonstrated high similarity in different metabolic assays of glucose uptake, adipogenesis activity, and inhibition of stimulated lipolysis. Rabbit bioassay studies showed MYL-1501D and EU-approved insulin glargine are highly similar to US-licensed insulin glargine. These product quality studies show high similarity between MYL-1501D and licensed or approved insulin glargine products and suggest the potential of MYL-1501D as an alternative cost-effective treatment option for patients and clinicians.

## Introduction

Insulin glargine is a long-acting analogue of human insulin that has been used to manage hyperglycemia in patients with diabetes mellitus (DM) for nearly 20 years [1, 2]. The relatively constant concentration-time profile of insulin glargine with no pronounced peak allows it to be administered on a once-daily basis and to mimic the basal levels of insulin required by patients with DM [3]. With a 90-minute onset of action and 24-hour duration of action, patients with type 1 DM (T1DM) or type 2 DM (T2DM) receiving insulin glargine may have lower risk of hypoglycemia, particularly nocturnal hypoglycemia, compared with intermediate-acting human insulin such as neutral protamine Hagedorn [3–5]. The delayed absorption and slower release of insulin glargine is due to its low aqueous solubility at neutral pH [3, 6]. In insulin glargine, asparagine at position 21 of the human insulin A-chain is replaced with glycine, and the carboxyl terminus of the insulin B-chain is elongated with the addition of 2 positively charged arginine molecules [3, 7]. The more positively charged amino acid sequence of insulin glargine causes a shift in the isoelectric point toward a neutral pH that, upon subcutaneous injection, creates a depot and a slower rate of absorption [7, 8].

Biosimilar formulations of reference products with proven long-standing clinical effectiveness are expected to deliver equivalent clinical effects at a lower cost to patients and society [9]. MYL-1501D (developed jointly by Viatris Inc, Canonsburg, PA, and Biocon Limited, Bangalore, India) was developed as a biosimilar/follow-on biologic to reference insulin glargine 100 U/mL (Lantus^®^; Sanofi-Aventis US LLC, Bridgewater, NJ). Therefore, MYL-1501D is also an analogue of human insulin made by replacing the asparagine at position 21 of the A-chain with glycine and adding 2 arginine residues to the C-terminus (positions 31 and 32) of the B-chain [10]. While MYL-1501D was designed to share primary and secondary structures with reference insulin glargine, the variability inherent in manufacturing biopharmaceuticals from living organisms, where “process is product,” may introduce differences between reference and follow-on products, which are evaluated in development and regulatory processes [11]. The potential for differences in immunogenicity between reference and follow-on insulin preparations is evaluated in comparative safety and immunogenicity clinical studies, as recommended by US and EU regulatory authorities [11–13]. Production of insulin glargine by the innovator uses a nonpathogenic strain of the prokaryote *Escherichia coli* [3] while MYL-1501D is expressed in the eukaryote (yeast) *Pichia pastoris* (also known as *Komagataella pastoris*), which generates some glycosylated forms of insulin glargine as product-related variants [10]. The manufacturing of MYL-1501D includes a purification process to remove the glycosylated forms of insulin glargine to ensure fidelity of purity, low risk of immunogenicity, and comparable functionality to the reference product. Potential differences between MYL-1501D and reference insulin glargine were evaluated through extensive physicochemical and functional characterization described herein. Following this characterization exercise, similarity of MYL-1501D to reference insulin glargine was concluded.

## Materials and methods

### Establishment of critical quality attributes

Critical quality attributes of MYL-1501D were defined to validate a control strategy in the production process that would yield indicators most relevant to clinical safety and efficacy for patients with DM. Criticality risk ranking was based on the principles of quality risk management published by the International Council on Harmonization (ICH) of Technical Requirements for Registration of Pharmaceuticals for Human Use [14, 15]. Since 1990, the ICH has brought industry and regulators together to increase quality and efficiency in the development of human medicines [16]. This process uses publicly available information about a molecular entity to rank the criticality of identifiable quality attributes. Similar tools have been used by the CMC Biotech Working Group to translate these principles to the development of biological entities, as exemplified by the case of a monoclonal antibody [17].

Quality attributes relevant to the safety and efficacy of insulin glargine were assessed and ranked using 2 risk ranking tools (ie, an impact and uncertainty tool and a severity and likelihood of severity tool). These tools help categorize the critical quality attributes of insulin glargine on a criticality continuum (“very high,” ‘high,” “moderate,” and “low) rather than as a binary classification of critical or non-critical. The risk ranking of insulin glargine quality attributes is summarized in S1 Table.

### Production and purification of MYL-1501D

The methylotrophic yeast *P pastoris* was selected for production of MYL-1501D because of its versatility and efficiency for mass production of heterologous proteins [18]. *P pastoris* is included in the list of biological agents with qualified presumption of safety status by the European Food Safety Authority [19]. As a eukaryote, notable advantages of *P pastoris* include its relatively rapid growth rate, amenability to molecular and genetic manipulation, and diverse posttranslational modifications such as protein processing and folding [20]. Additionally, *P pastoris* lacks endotoxin contamination that is typical of *E coli*–based expression systems, and *P pastoris* is not conducive to amplification of viruses that are infectious in humans [20, 21]. *P pastoris* does, however, generate multiple low-molecular-weight glycosylated forms of recombinant protein as product-related variants.

Seed culture preparation for MYL-1501D involves the inoculation of seed flasks from a qualified working cell bank culture prepared using a recombinant yeast strain of *P pastoris* (strain GS115; Invitrogen Corporation, Carlsbad, CA) carrying a gene encoding the insulin glargine precursor. The seed flasks are incubated at predefined conditions and transferred to inoculum flasks until the culture meets the defined growth criteria. The inoculum flasks are pooled and transferred to a seed fermenter to increase cell mass. Fermentation is then carried out in a production fermenter in the following 2 phases: the batch phase increases cell mass, and the fed-batch phase uses methanol to induce secretion of the insulin glargine precursor into the medium. Once secreted, the insulin glargine precursor is isolated and converted to insulin glargine in the downstream process.

To ensure similar product quality of MYL-1501D to reference insulin glargine, a 2-stage purification process was developed to selectively eliminate all glycosylated variants of the insulin glargine preparation to below the limit of quantitation. Stage 1 (downstream-I) separates out the insulin glargine precursor and converts it to insulin glargine. First, the supernatant from the fermentation process containing the insulin glargine precursor is clarified using a pH-based precipitation followed by centrifugation. It is then loaded onto a cation exchange chromatography column to allow for the capture and concentration of the insulin glargine precursor, which is converted to insulin glargine in a trypsin-catalyzed enzyme reaction. The converted insulin glargine is precipitated and centrifuged. Stage 2 (downstream-II) purifies insulin glargine from process- and product-related impurities that were generated during both fermentation and the enzyme reaction by using reverse-phase high-performance liquid chromatography (RP-HPLC) [22].

### Establishment of comparability with the reference product

MYL-1501D is commercially sold by Mylan and can be acquired similarly to the innovator product, ie, Lantus^®^, through global drug sourcing companies such as Myonex (formerly Myoderm; Norristown, PA, USA). Multiple lots of MYL-1501D and US-licensed insulin glargine (Lantus^®^ Insulin 100 U/mL 10-mL vial, NDC 0008-2220-33; Lantus^®^ Solostar^®^ 100 U/mL 3-mL prefilled pen, NDC 00088-2219-05) and EU-approved insulin glargine (Lantus 3 mL × 5 cartridges, MA/EU license EU/1/00/134/006) preparations were acquired from Myonex. In the United States, MYL-1501D is available as Semglee^®^ insulin glargine (100 U/mL 10-mL vial, NDC 49502-195-80) and in the European Union as Semglee^®^ 3 mL × 5 cartridges (MA/EU license number EU/1/18/1270/003). MYL-1501D and US-licensed and EU-approved insulin glargine were used to analyze protein structure, product-related variants, and in vitro functional activity among the follow-on and reference products.

#### Statistical evaluation to establish analytical similarity

To confirm the equivalence of 2 drugs, classical hypothesis testing is not considered suitable and use of equivalence testing is preferred. Two One-Sided T-test (TOST/Equivalence test) is used to validate the equivalence of 2 means, which primarily confirms equivalence. In a classical hypothesis test (*t* test or ANOVA), the null hypothesis of equality is rejected; however, equivalence tests validate the equivalence between 2 samples. TOST is a test of equivalence that is based on the classical *t* test but evaluates the hypothesis of equality between 2 means. The *P* value is generated for each of the assessments and a *P* value of <0.05 signifies the establishment of equivalence.

Analytical similarity is considered demonstrated if the 90% 2-sided confidence interval of the mean difference is within the defined equivalence margin. The equivalence margin is a function of the variability of the reference product as established from the innovator reference product lots analyzed.

Additionally, a quality range (QR) approach was also used to confirm the comparability of MYL-1501D to US- and EU-licensed insulin glargine. The QR limits were set based on the range of the values obtained for reference product variation, expressed as 3 times standard deviation (SD), or mean ± 3 SD. Comparability was considered demonstrated if 90% of MYL-1501D lot values fell within the quality range as established from the innovator reference product lots analyzed (US- and EU-licensed insulin glargine).

### Liquid chromatography–mass spectrometry (LC-MS) analysis: Intact, reduced, and non-reduced peptide mapping

Reverse-phase high-performance liquid chromatography–mass spectrometry (RP-HPLC-MS)-based assays were used to determine the amino acid sequence, thus confirming the primary protein structure, of MYL-1501D and US- and EU-licensed insulin glargine samples by assessing the masses of intact, reduced, and digested peptides.

#### Intact and reduced mass analysis

Reverse-phase HPLC followed by electrospray ionization LC-MS (LC-ESI-MS; 5 μ C18-300Å 250 × 4.6 mm) determined the intact and reduced masses of the drug product samples. Chromatographic separation of samples occurred using a reverse-phase C8 column (5 μ C8 250 × 4.6 mm), which was simultaneously ionized to give a mass to charge ratio (m/z) for particular species. Mass of intact insulin glargine was determined using untreated protein samples. Reduced masses of the insulin A- and B-chains were obtained via dithiothreitol (DTT)-mediated reduction of disulfide bonds.

#### Peptide mass fingerprinting

MYL-1501D and reference product samples were digested with glutamyl endoproteinase Glu-C (Cat No: 11047817001, Roche, Basel, Switzerland). Disulfide bonds were reduced by incubating for an additional hour in 1 M of DTT (Cat No: RO861, Thermo Scientific, Waltham, MA). DTT-treated samples were used in reduced peptide mapping, whereas the non-reduced samples provided information on disulfide mapping. Individual peptide fragments were identified using LC-MS. Detection was performed using UV radiation at 215 nm; the Orbitrap XL mass spectrometer (ThermoFisher Scientific) was set to a resolution of 30,000, with a capillary voltage of +5.5 kV, tube lens of 100 V, collision energy of 35 V, mass range of m/z 100 to 2000 Da (for MS and MS/MS), and source temperature of 330°C.

### Circular dichroism (CD) spectroscopy

Near (260–360 nm) and far (190–260 nm) UV circular dichroism (UV-CD) spectroscopy was used to study tertiary and secondary protein structures, respectively. Circular dichroism experiments were carried out on a Jasco J-815 spectrometer equipped with a Peltier-type cell holder. Spectra were recorded at 25°C with a scanning speed of 200 nm/min and a bandwidth of 0.1 nm in a quartz cell, with a path length of 0.1 cm. Far-UV spectra were recorded from 190 to 260 nm at a sample concentration of 0.3 mg/mL, whereas near-UV spectra were recorded from 260 to 360 nm at a sample concentration of 3 mg/mL. For each sample, 6 accumulations were performed, and baseline correction was applied.

### Nuclear magnetic resonance (NMR) spectroscopy

Nuclear magnetic resonance spectroscopy was performed on drug substance extracted from MYL-1501D and US- and EU-licensed insulin glargine preparations. Drug products were loaded onto a chromatography column using an injection volume in the range of 15% to 25% of the column volume. Protein collection began when UV absorbance at 280 nm reached 10 mAU, and collection stopped after UV absorbance at 280 nm began to decrease. Samples of MYL-1501D and reference products were lyophilized and prepared for NMR by dissolving 4 mg in 400 μL of 60% miliQ water combined with 40% acetonitrile-d3 (pH 4.0; product number: 151807, Sigma-Aldrich); a 0.01-N hydrochloric acid solution (Cat No: 339253, Sigma-Aldrich, St Louis, MO) was used to adjust the pH. A Bruker Avance III spectrometer operating at field strength of 500.15 MHz equipped with a 5-mm TCI CryoProbe (ICGEB, New Delhi, India) was used; probe temperature was maintained at 27°C, and the sample tubes were not centrifuged. The following NMR experiments were performed:

1. 1D 1H: t_1max_ = 4.09 s, spectral width (SW) (1H) = 8012.82 Hz
2. 2D [1H, 1H] total correlated spectroscopy (TOCSY) with 60 ms mixing time
3. 2D [1H, 1H] nuclear Overhauser effect spectroscopy (NOESY) with 100 and 250 ms mixing time

Where t_1max_ = 36.56 ms, t_2max_ = 146.28 ms, and SW (1H) = 7002.8 Hz for both 2D NOESY and 2D TOCSY.

The reference standard was obtained by measuring the isolated peak belonging to the 1-HN proton of cysteine-A11 at δ = 9.39 ppm1 in the 1D 1H spectrum of insulin and adjusting the chemical shift of the corresponding peak in the 2D spectra to the same value. NMR data were processed with TopSpin™ 3 software (Bruker, Billerica, MA) using 2048 D1 and 1024 D2 data points. A 90° shifted sin2 bell function was used for resolution enhancement. Third-order polynomial baseline corrections were used in both dimensions (D1 and D2), particularly for in-phase NMR data. CARA software (Wüthrich Group, ETH Zürich, Zürich, Switzerland) was used for all NMR analyses and figure preparation.

### Fourier-transformed infrared (FTIR) spectroscopy

Fourier-transformed infrared spectroscopy was used for structure mapping. All measurements were performed with an attenuated total reflection attachment on a JASCO/FTIR-6300 type A spectrometer. The IR spectra were recorded between 4000 cm^-1^ and 600 cm^-1^, with an 8-wavenumber resolution at a concentration of 25 to 30 mg/mL. Spectral analysis was performed using Spectra Manager CFR^®^ (Jasco, Oklahoma City, OK). Each spectrum was averaged over 256 scans and baseline corrected.

### Differential scanning calorimetry (DSC)

Thermal stability measurements were performed using a Nano DSC differential scanning calorimeter (TA Instruments, New Castle, DE). Samples were scanned from 20°C to 100°C, and scans were recorded at a rate of 1.5°C/min. Independently, a placebo-containing polysorbate 20 was run as a control and was subtracted for the T_M_ analysis.

### X-ray crystallography

X-ray crystallography studies were performed on drug substance extracted from MYL-1501D and US- and EU-licensed insulin glargine preparations. Samples were loaded onto a chromatography column using an injection volume in the range of 15% to 25% of the column volume. Protein collection began when UV absorbance at 280 nm reached 10 mAU and stopped after UV absorbance at 280 nm began to decrease. The pH of the samples was adjusted to 7.0 using 1.5 M of Tris-hydrochloric acid buffer (Cat No: 10812846001, Roche Diagnostics GmbH). The final concentration of the protein sample was adjusted to approximately 9.0 mg/mL, and this sample was used in the 2 crystallization experiments.

Crystallization experiments were performed using a microbatch-under-oil method. Crystallization solutions for the samples were as follows: 0.2 M of sodium citrate tribasic dehydrate (Cat No: S4641, Sigma-Aldrich), 0.1 M of Tris-hydrochloric acid pH 8.5 (Cat No: 10812846001, Roche Diagnostics GmbH), and 30% v/v polyethylene glycol 400 (Cat No: 8074855000, Sigma-Aldrich) and for the sample labeled 5F193A, 0.2 M of sodium citrate tribasic dehydrate (Cat No: S4641, Sigma-Aldrich), 0.1 M of Tris-hydrochloric acid pH 8.5, and 30% v/v polyethylene glycol 400. In both experiments, crystallization drops were composed of 1.0 μL of insulin glargine sample and 1.0 μL of crystallization solution. Crystals for both samples were observed within 48 hours. Diffraction data were collected on an R-Axis IV++ detector (Rigaku, Tokyo, Japan) mounted on an FRE x-ray generator (Rigaku) set at 45 kV and 55 mA (IISc, Bangalore, India). Complete x-ray diffraction data sets on both crystals were collected using screenless oscillation photography. Based on the initial diffraction pattern, the exposure time and crystal oscillation angles were set to 1.0 min and 1°, respectively. The crystal-to-detector distance was kept at 120 mm. Crystals from both samples belonged to the space group I23. In both cases, the crystal asymmetric unit contained 1 insulin molecule (solvent content, 47%). Data sets were processed using the MOSFLM suite of programs, and resulting intensities were scaled using SCALA (CCP4 suite).

### Intrinsic fluorescence

Intrinsic fluorescence measurements were performed on a Cary Eclipse fluorescence spectrometer (Agilent Technologies), and data were analyzed using Scan software version 1.2 (Agilent Technologies, Santa Clara, CA). Intrinsic fluorescence analysis was performed by excitation at 278 nm, and emission was scanned from 300 to 400 nm.

### Analytical ultracentrifugation (AUC)

Analytical ultracentrifugation was used to characterize aggregates and fragments in terms of heterogeneity and particle size. MYL-1501D and reference products were loaded into cells with 2-channel charcoal-epon centerpieces and a 12-mm optical path length. Dilution buffer was loaded into the reference cell to nullify any effects due to excipients in the spectrophotometer. Loaded cells were placed into an AN-60Ti analytical rotor (Beckman Coulter, Brea, CA), loaded into a Proteome Lam XL-A analytical ultracentrifuge (Beckman Coulter), and brought to 20°C. When the rotor reached 3000 rpm, the samples were scanned at 230 nm to confirm proper cell loading before the rotor reached a final run speed of 60,000 rpm. Scans were recorded every 4 minutes for approximately 4 hours (approximately 50 scans per sample); the scan rate was then dropped to every 16 minutes for an additional 8 hours.

Data were analyzed using SEDFIT software version 11.3 [23]. Sedimentation coefficients were derived by fitting the raw data while modeling the influence of diffusion. Briefly, a diffusion coefficient was assigned to each value of sedimentation coefficient based on the assumption that all species have the same overall hydrodynamic shape, where the shape was defined by the frictional coefficient ratio relative to sphere, f/f_0_. The f/f_0_ values were varied to find the best overall fit of the data for each sample. A maximum entropy regularization probability of 0.683 (1 σ) was used, and time-invariant noise was removed. The resultant size distributions were graphed, and the peaks were integrated using Origin software (OriginLab Version 2018b).

### Size-exclusion high-performance liquid chromatography (SE-HPLC)

The purity of MYL-1501D and reference preparations was analyzed using SE-HPLC. Size variant separation for resolution of monomers and aggregates was performed using an HPLC connected with Waters Insulin high-molecular-weight protein (HMWP) column (7.8 × 300 mm; Waters, Milford, MA) at 25°C. HMWP mobile buffer was prepared as a solution of L-arginine (1 g/L; Cat No: A5006, Sigma-Aldrich), acetic acid (Cat No: 695092, Sigma-Aldrich), and acetonitrile (Cat No: 9017–03, J.T. Baker, Phillipsburg, NJ) in a ratio of 65:15:20. Isocratic elution was carried out at a flow rate of 0.5 mL/min, with monitoring and detection of peaks at UV 276 nm; total run time was 35 minutes. Samples of MYL-1501D and reference products were prepared at a concentration of 4.0 mg/mL in 0.01 N of hydrochloric acid.

### Metabolic and mitogenic activity *in vitro* and *in vivo*

#### Insulin receptor (IR)-A (short-form) and IR-B (long-form) binding kinetics assay

Surface plasmon resonance was used to evaluate the binding of MYL-1501D and reference insulin glargine samples to purified recombinant IR-A or IR-B using a Biacore T100 instrument (GE Healthcare Bio-Sciences, Uppsala, Sweden). Briefly, IR-A or IR-B was immobilized on the surface of a CM5 sensor chip, and the MYL-1501D and reference product samples were passed over the surface. Accumulation of biosimilar or reference insulin glargine as it binds IR-A or IR-B results in an increase in the refractive index. A sensorgram was plotted to visualize this change in refractive index, which was measured in real time as response or resonance units (RU) vs time. Binding affinity was determined in terms of rate of association (k_a_), rate of dissociation (k_d_), and dissociation constant (K_D_) using a 1:1 interaction binding model.

Before sample analysis, the Biacore T100 instrument was washed with 50 mM of NaOH (Cat No: 20278961791, GE Healthcare) to regenerate the CM5 chip. The blank/reference cells were activated with the coupling agents 1-ethyl-3-(3-dimethylaminopropyl) carbodiimide (EDC) and N-hydroxy succinimide (NHS; Cat No: 2076029, GE Healthcare) amine, followed by blocking with ethanolamine (Cat No: 2076029, GE Healthcare). In the sample cells, IR-A or IR-B was coupled to the CM5 binding surface by first washing the cells with EDC/NHS, followed by injection of 6 μg/mL of IR-A or IR-B. IR-A or IR-B was immobilized on the CM5 chip in 10 mM of acetate buffer to a baseline of 1500 RU.

Samples of MYL-1501D and reference products were prepared in HBS-EP+ buffer (Cat No: BCBP7344V, GE Healthcare) and analyzed at concentrations of 0.003125, 0.00625, 0.0125, 0.025 (run-in duplicate), 0.05, and 0.1 μM. Association of the insulin receptor with the sample preparations in buffer occurred at a flow rate of 30 μL/min for 150 seconds, followed by dissociation with buffer alone for 200 seconds. Samples were analyzed with BIAevaluation software (Biacore, Uppsala, Sweden) using the same template and constant curve fit with a 1:1 Langmuir binding model and RI constant set to 0.

#### Insulin growth factor-1 receptor (IGF-1R) binding kinetics assay

The Biacore T200 instrument was prepared by washing the CM5 chip with 50 mM of NaOH. All flow cells were activated for amine coupling with EDC/NHS, after which reference flow cells were blocked with ethanolamine, and sample cells were loaded with 5.5 μg/mL of IGF-1R protein in 10 mM of acetate buffer (Cat No: BR-1003-49, GE Healthcare).

Samples of MYL-1501D and reference products were diluted in HBS-EP buffer (Cat No: BR-1001-88, GE Healthcare) to concentrations of 3.00, 1.50, 0.75, 0.38 (run-in duplicate), 0.19, and 0.09 μM. Running buffer was processed through the instrument for 5 start-up cycles, followed by addition of the diluted samples in buffer at a flow rate of 50 μL/min maintained for 60 seconds for association before dissociation with buffer alone for 150 seconds. Kinetic run data traces were evaluated using a 1:1 Langmuir model with RI set to 0.

#### IR-A and IR-B phosphorylation in CHO-K1 cells

Recombinantly engineered CHO-K1 cells (Trenzyme GmbH, Konstanz, Germany) overexpressing IR-A or IR-B were seeded at 25,000 cells/well for IR-A phosphorylation and 10,000 cells/well for IR-B phosphorylation in 100 μL of serum-free, incomplete DMEM growth medium (Cat No: 10565, Gibco) in a 96-well plate for 22 to 24 hours at 37°C and 5% CO_2_. Medium was replaced with 100 μL of incomplete medium containing the following concentrations of MYL-1501D or reference insulin glargine preparations: 2.34, 4.69, 9.38, 18.75, 37.50, 75.00, 150.00, and 300.00 ng/mL (IR-A phosphorylation) and 3.13, 6.25, 12.50, 25.00, 50.00, 100.00, 200.00, and 400.00 ng/mL (IR-B phosphorylation). After 5 to 7 minutes of incubation at 37°C in a 5% CO_2_ incubator, cells were washed with ice-cold Dulbecco’s phosphate-buffered saline (DPBS; Cat No: 14190, Gibco) and lysed in 50 μL of lysis buffer. Then, 12 μL of the solution were transferred to a white, half-area 96-well plate and mixed with 15 μL/well of acceptor mix from the AlphaScreen^®^ SureFire^®^ Insulin Receptor kit (Cat No: TGRIRS10K, Perkin Elmer, Waltham, MA) for 2 hours at room temperature. After a final incubation with the donor mix for 2 hours, the plate was read at an excitation of 680 nm and emission of 615 nm. Concentration-response data were analyzed using Four Parameter Logistic (4PL) Regression (SoftMax Pro, Molecular Devices, San Jose, CA) and Parallel Line Analysis (PLA) software (Stegmann Systems GmbH, PLA 3.0, Rodgau, Germany) and compared with working standard QC/Q3/WS-EP/006/05.

#### Total IR phosphorylation in HepG2 cells

A 96-well plate was seeded with 100,000 cells/well in 100 μL of complete RPMI growth medium (Cat No: 11875, Gibco) and incubated at 37°C in a 5% CO_2_ incubator for 22 to 24 hours. After incubation, the cells were washed with DPBS, and the medium was replaced with serum-free, incomplete medium and further incubated for 22 to 24 hours. After the second incubation, incomplete medium was replaced with 100 μL of fresh incomplete medium containing the following concentrations of MYL-1501D or reference insulin glargine preparations: 8.40, 14.00, 23.33, 38.88, 64.80, 108.00, 180.00, 300.00, and 500.00 ng/mL. After a 5- to 7-minute incubation with the study drug, the cells were washed with ice-cold DPBS and lysed in 50 μL of lysis. Then, 12 μL of the cell lysate were transferred to a new, white 96-well plate and incubated for 2 hours with 15 μL/well of acceptor mix from the AlphaScreen SureFire Insulin Receptor kit. Next, 6 μL/well of donor mix were added and incubated for 2 hours, at which time the plate was read with an excitation at 680 nm and emission at 615 nm. Concentration-response data were analyzed using PLA software and compared with working standard QC/Q3/WS-EP/006/05.

#### Glucose uptake/consumption in 3T3-L1 cells

Glucose uptake/consumption studies used the glucose oxidase/peroxidase assay to measure the ability of insulin glargine preparations to stimulate glucose uptake in differentiated murine 3T3-L1 adipocyte cells. Briefly, 3T3-L1 cells (Cat No: CL-173, ATCC, Manassas, VA) were seeded at a density of 25,000 cells/well in 200 μL of complete L1 medium (Cat No: 11995, Invitrogen) in a 96-well plate and incubated for 70 ± 2 hours at 37°C and 5% CO_2_. The medium was replaced with 200 μL of differentiation medium, incubated for 94 ± 2 hours at 37°C and 5% CO_2_ and then replaced with 200 μL of complete L1 medium and further incubated for 70 ± 2 hours at 37°C and 5% CO_2_. During this incubation, drug doses were prepared at a 2× concentration in low-glucose assay medium (Cat No: D-5921, Sigma-Aldrich) to be diluted to final concentrations of 1.56, 3.13, 6.25, 12.50, 25.00, 50.00, 100.00, and 200.00 ng/mL. After the 70-hour incubation in L1 medium was complete, the medium was removed, cells were rinsed with DPBS, and the medium was replaced with 100 μL of DPBS and 100 μL of the diluted insulin glargine concentrations in low-glucose medium. Cells were incubated for 22 ± 2 hours at 37°C and 5% CO_2_; then, 10 μL of cell culture supernatant were transferred to a fresh, clear 96-well plate, and 85 μL of water, 40 μL of peroxidase substrate, and 65 μL of glucose oxidase/peroxidase reagent mix (Cat No: G-3660, Sigma-Aldrich) were added. A final incubation at 37°C for 10 ± 2 minutes was followed by reading absorbance at 550 nm in a spectrophotometer.

#### Adipogenesis in 3T3-L1 cells

Cells were seeded in 96-well plates at a density of 30,000 cells/well in 100 μL of pre-adipocyte medium and were incubated for 48 hours in a humidified incubator at 37°C and 5% CO_2_. The pre-adipocyte medium was aspirated, and 75 μL of adipocyte maintenance medium (Cat No: AM-1-L1-IF, ZenBio, Research Triangle Park, NC), which lacked insulin but contained 3-isobutyl-1-methylxanthine (IBMX; Cat No: PT-9013H, Lonza, Basel, Switzerland), were added to the plates. On day 3, adipogenesis was carried out using samples of MYL-1501D and US- and EU-licensed insulin glargine; 8-point dilutions were prepared in adipocyte maintenance medium on day 1 of differentiation. The 2× dilutions made were added in triplicate for a total well volume of 150 μL, and the plates were incubated at 37°C and 5% CO_2_ for an additional 6 days. On day 7 of differentiation, the medium from the plates was removed, 100 μL of lipid extraction buffer (Cat No: K610-100, BioVision, Milpitas, CA) were added to the wells, and the plates were incubated on a shaker at 90°C and 70 rpm for 30 minutes.

The plates were used as per the assay procedure described in the BioVision kit protocol (Cat No: K610-100, BioVision). The plates were read at excitation/emission wavelengths of 535/590 nm on EnVision (Perkin Elmer). Relative potency (100%) of standard run in every plate was calculated using SoftMax Pro 5.4.1 software.

#### Inhibition of stimulated lipolysis in 3T3-L1 cells

Cells were seeded at a density of 30,000 cells/well in 100 μL of pre-adipocyte medium in a 96-well plate and incubated for 48 hours in a humidified incubator at 37°C and 5% CO_2_. On day 3, differentiation medium containing IBMX plus dexamethasone plus 0.1 μM of rosiglitazone plus insulin was added to the 96-well plate and incubated for 3 days in a humidified incubator at 37°C and 5% CO_2_. On day 6, the medium was replaced with adipocyte maintenance medium without dexamethasone and incubated for an additional 3 days in a humidified incubator at 37°C and 5% CO_2_. On day 10, minimum essential medium alpha starvation was performed overnight with 1 nM of human insulin (Cat No: I9278, Sigma-Aldrich). Cells were treated with increasing concentrations of the MYL-1501D and reference insulin glargine preparations in Krebs-Ringer bicarbonate-pyruvate medium (Cat No: K612-100, BioVision) containing 1% BSA for 1 hour, followed by stimulation with 3 nM of isoproterenol (Cat No: I5627, Sigma-Aldrich) for 2 hours. Supernatant was collected, a free fatty acid assay was performed (Cat No: K612-100, BioVision), and the plate was read for absorbance at 570 nm. Inhibition of stimulated lipolysis was calculated using the ratio of half-maximal effective concentration values from the test samples vs the working standards in murine 3T3-L1 cells.

#### Mitogenesis/proliferation assay in sarcoma osteogenic (Saos-2) cells

Additional mitogenic activity was investigated according to proliferation of Saos-2 (Cat No: HTB-85, ATCC) cells exposed to insulin glargine preparations. Briefly, Saos-2 cells were trypsinized and seeded at 40,000 cells/100 μL/well in Saos-2 assay medium (Cat No: AL057A, HiMedia, Mumbai, India) in a 96-well plate and incubated for 24 hours at 37°C and 5% CO_2_. After 24 hours, the cell culture medium was removed and replaced with medium containing MYL-1501D or reference insulin glargine samples at final concentrations of 20, 5, 1.25, 0.3125, 0.0781, 0.0195, 0.0049, and 0.0012 μg/mL; control wells included cells alone or cell culture medium containing 0.5 μg/mL of IGF-1 or 0.5 μg/mL of VEGF. Cells were incubated for 96 hours at 37°C in a 5% CO_2_ incubator before addition of 40 μL of Alamar Blue reagent (Cat No: DAL1100, Invitrogen) to each well and incubation for 3.5 ± 0.5 hours at 37°C and 5% CO_2_. After incubation, fluorescence at 530 nm excitation and 590 nm emission was read in a Synergy plate reader. Relative fluorescence units were used to plot and analyze mitogenesis using PLA software.

#### Rabbit bioassay of *in vivo* insulin glargine activity

The most predominant effect of insulin activity is a sudden decrease in blood glucose and this effect forms the basis for the potency assay in rabbits. The relative potency of MYL-1501D and reference insulin glargine products was compared with the US Pharmacopeia (USP) reference standards using an *in vivo* rabbit bioassay (USP Chapter <121>). Twenty-four rabbits were divided into 4 groups of 6 each. The rabbits were injected subcutaneously with standard solution (USP insulin glargine) and test solution (MYL-1501D or insulin glargine) on days 1 and 3. Plasma glucose levels were measured using automatic analyzer (Rx Daytona clinical chemistry analyzer) at 1 and 2.5 hours after the day 1 and day 3 injections. Glucose values were subjected to statistical analysis as recommended in USP <121> and USP <111> to estimate the potency of the test samples. Acceptance criteria required MYL-1501D and reference product potency to fall within 10% of the published insulin glargine and the confidence interval of the log relative potency should be greater than 0.082, which corresponds at *P* = 0.95. The assay was repeated, and the results were combined and assessed as per USP <111> until the relative potency met the acceptance criteria. USP human insulin standard Lot JOJ250 (potency, 26.40 U/mg) and USP insulin glargine standard Lot F009M0 (27.49 U/mg) were used [24].

## Results

### Physicochemical characterization of insulin glargine preparations

Detailed structural characterization of MYL-1501D, US-licensed insulin glargine, and EU-licensed insulin glargine was performed using state-of-the-art analytical techniques (Table 1), which provided information on primary, secondary, and higher-order structures, as well as protein content, product-related impurities, and product-related substances. Table 2 presents a summary of the results of the physicochemical characterization of the 3 products.

**Table 1:** Similarity testing plan, analytical methods and product characteristics for the structural and functional characterization.

Quality attribute	Analytical method	Product characteristics/variants
Primary structure		
Intact mass	LC-ESI-MS	6063.9 (± 1 Da)
Reduced mass of A and B chains		Chain A: 2326.8 (± 1 Da)
		Chain B: 3743.1 (± 1 Da)
Primary sequence	Peptide mass fingerprinting, reduced	Fragment 1: 456.1 (± 1 Da)
		Fragment 2: 417.23 (± 1 Da)
		Fragment 3: 1482.7 (± 1 Da)
		Fragment 4: 1428.8 (± 1 Da)
		Fragment 5: 867.4 (± 1 Da)
		Fragment 6: 1490.6 (± 1 Da)
Secondary structure		
Ellipticity determination	Far UV-CD spectroscopy	α Helix: 21.3%-36.0%
		β sheet + β turn: 47.6%-54.4%
		Random coil: 12.9%-27.8%
Position of amide I and II	FTIR spectroscopy	Amide I: 1647.9 ± 1.5 cm^-1^
		Amide II: 1538.1 ± 1.5 cm^-1^
Tertiary structure		
Establishing disulfide linkages	Peptide mass fingerprinting, non-reduced	Cluster 1 {B(14–21)}—{A(l8-21)}: 1320.5 (± 1 Da)
		Cluster 2 {B(1–13)}—{A(5–17)}: 2969.4 (± 1 Da)
	Near UV-CD spectroscopy	
Environment of aromatic amino acids		
		Spectral comparison
Environment of intrinsic chromophores in folded structure	Fluorescence spectroscopy	301 nm (± 2 nm)
Solution phase conformation of protein structure	NMR spectroscopy	Disulfide confirmation, spectral comparison
Atomic resolution model to confirm structural integrity	X-ray diffraction	RMSD, disulfide confirmation, number of outliers
	Differential scanning calorimetry	
Thermal stability of insulin		Thermal unfolding temperature (T_M_ °C)
Product-related variants		
Purity and related substances	RP-HPLC	USP limits for DS batch release:
		A21: NMT 2%
		ORP: NMT 2%
HMWP	SEC-UV	Qualitative estimate of HMWP:
		DS: ≤1%
		R: ≤1.7%
Product attributes		
Protein content	RP-HPLC	Quantitative estimate of content and purity:
		DS: NLT 27.5 unit/mg
		DP: 100 unit/mL, 95.0%-105.0%
Zinc content	Atomic absorption spectroscopy	Quantitative estimation of zinc content:
		R: 10–40 μg/100 IU
Functional assays: metabolic activity		
Insulin-induced adipogenesis	Adipogenesis assay in 3T3-L1 cells	The %CV values for relative potency should be ≤25%
Insulin-induced inhibition of stimulated lipolysis	Inhibition of stimulated lipolysis assay in 3T3-L1 cells	The %CV values for relative potency should be ≤25%
Cell-based total insulin receptor phosphorylation	AlphaScreen^®^ Surefire^®^ technology	The %CV values for relative potency should be ≤25%
IR-B (long-form) binding kinetics	SPR (Biacore)	The %CV values for k_a_, k_d_, K_D_, and R_max_ should be ≤25%
Cell-based IR-B phosphorylation	AlphaScreen Surefire technology	The %CV values for relative potency should be ≤25%
Glucose uptake/consumption assay	Glucose uptake/consumption assay in 3T3-L1 cells	The %CV values for relative potency should be ≤25%
Functional assays: mitogenic activity		
IGF-1R binding kinetics	SPR (Biacore)	The %CV values for k_a_, k_d_, K_D_, and R_max_ should be ≤25%
Mitogenic potential of insulin	Cell-based assay to study Saos-2 cell proliferation	The %CV values for relative potency should be ≤25%
Cell-based IR-A phosphorylation	AlphaScreen Surefire technology	The %CV values for relative potency should be ≤25%
IR-A (short-form) binding kinetics	SPR (Biacore)	The %CV values for k_a_, k_d_, K_D_, and R_max_ should be ≤25%

CV, coefficient of variation; DP, drug product; DS, drug substance; FTIR, Fourier-transformed infrared; HMWP, high-molecular-weight protein; IGF-1R, insulin-like growth factor-1 receptor; IR-A, insulin receptor A; IR-B, insulin receptor B; k_a_, rate of association; k_d_, rate of dissociation; K_D_, dissociation constant; LC-ESI-MS, liquid chromatography electrospray ionization–mass spectrometry; NLT, not less than; NMR, nuclear magnetic resonance; NMT, not more than; ORP, oxidation-reduction potential; R_max,_ maximum response; RMSD, root mean square deviation; RP-HPLC, reverse-phase high-performance liquid chromatography; SEC-UV, size-exclusion chromatography–ultraviolet absorption; SPR, surface plasmon resonance; USP, United States Pharmacopeia; UV-CD, ultraviolet–circular dichroism.

**Table 2:** Structure and purity/variant physicochemical studies of MYL-1501D and US and EU reference products.

Analytical method	Quality attribute	Minimum–maximum observed range (number of lots tested)		
		MYL-1501D	EU reference product	US reference product
**1. Structural measures**				
Far UV-CD	α-Helix, %	26.6–30.2 (10)	20.0–27.1 (22)	18.7–28.7 (22)
	β-Sheet, %	45.0–49.9 (10)	29.5–49.8 (22)	33.1–54.1 (22)
	β-Turn, %	6.2–9.9 (10)	7.5–21.0 (22)	5.9–18.8 (22)
	Random coils, %	15.0–17.5 (10)	16.9–25.9 (22)	17.9–22.9 (22)
FTIR	α-Helix, %	23.0–31.0 (10)	23.0–34.0 (22)	22.0–33.0 (22)
	β-Sheet, %	23.0–31.0 (10)	19.0–31.0 (22)	20.0–33.0 (22)
	β-Turn, %	21.0–22.0 (10)	21.0–22.0 (22)	21.0–23.0 (22)
	Random coils, %	23.0–24.0 (10)	23.0–25.0 (22)	23.0–25.0 (22)
DSC	Temperature, °C	70.2–73.9 (10)	65.9–74.0 (10)	68.4–73.5 (10)
**2. Purity/Variant measures**				
SE-HPLC	HMWP, %	≤0.06 (10)	≤0.06 (22)	≤0.06 (22)
SEC-MALS	Mn, kDa	5.53–5.69 (10)	5.46–5.77 (10)	5.58–5.70 (10)
	Mw, kDa	5.56–5.71 (10)	5.48–5.78 (10)	5.59–5.72 (10)
	Mz, kDa	5.59–5.73 (10)	5.50–5.80 (10)	5.60–5.73 (10)
	Mw/Mn	1.00–1.01 (10)	1.00–1.01 (10)	1.00–1.01 (10)
	Mz/Mn	1.00–1.01 (10)	1.00–1.01 (10)	1.00–1.01 (10)
AUC	Monomer sedimentation coefficient, S	1.58–1.65 (11)	1.62–1.68 (5)	1.61–1.64 (8)
	Total aggregate fraction, %	0.0–3.9 (11)	0.0–3.6 (5)	0.0–3.2 (8)
RP-HPLC	Protein content, mg/mL	3.48–3.71 (10)	3.48–3.81 (22)	3.46–3.90 (22)
	Overall content, %, U/mL	95.8–102.1 (10)	95.6–104.6 (22)	95.0–107.2 (22)

Summary of minimum and maximum observed ranges for structural attributes and purity/variants analyzed for multiple lots of MYL-1501D and US and EU reference products using a suite of analytical methods. The number of lots analyzed for each product is presented in parentheses.

AUC, analytical ultracentrifugation; DSC, differential scanning calorimetry; FTIR, Fourier-transformed infrared spectroscopy; HMWP, high-molecular-weight protein; Mn, number-average molecular weight; Mw, weight-average molecular weight; Mz, z-average molecular weight; RP-HPLC, reverse-phase high-performance liquid chromatography; S, Svedbergs; SE-HPLC, size-exclusion HPLC; SEC-MALS, SE chromatography with multiangle light scattering; UV-CD, UV–circular dichroism.

### Primary structure analysis and molecular weight

Peptide mapping and MS/MS analysis confirmed that the primary amino acid sequence of MYL-1501D was identical to the sequences of US- and EU-licensed insulin glargine. Intact mass analysis confirmed identical average molecular masses of intact MYL-1501D and reference insulin glargine products when considering mass accuracy of ± 1 Da (Table 1). Upon reduction with DTT, the reduced mass analysis showed identical average molecular weights of the A and B chains within the mass accuracy of ± 1 Da (Table 1). The average molecular masses obtained from these experiments were very consistent with the theoretical masses of the intact insulin glargine and the corresponding A and B chains for the 3 products.

**Fig 1:**
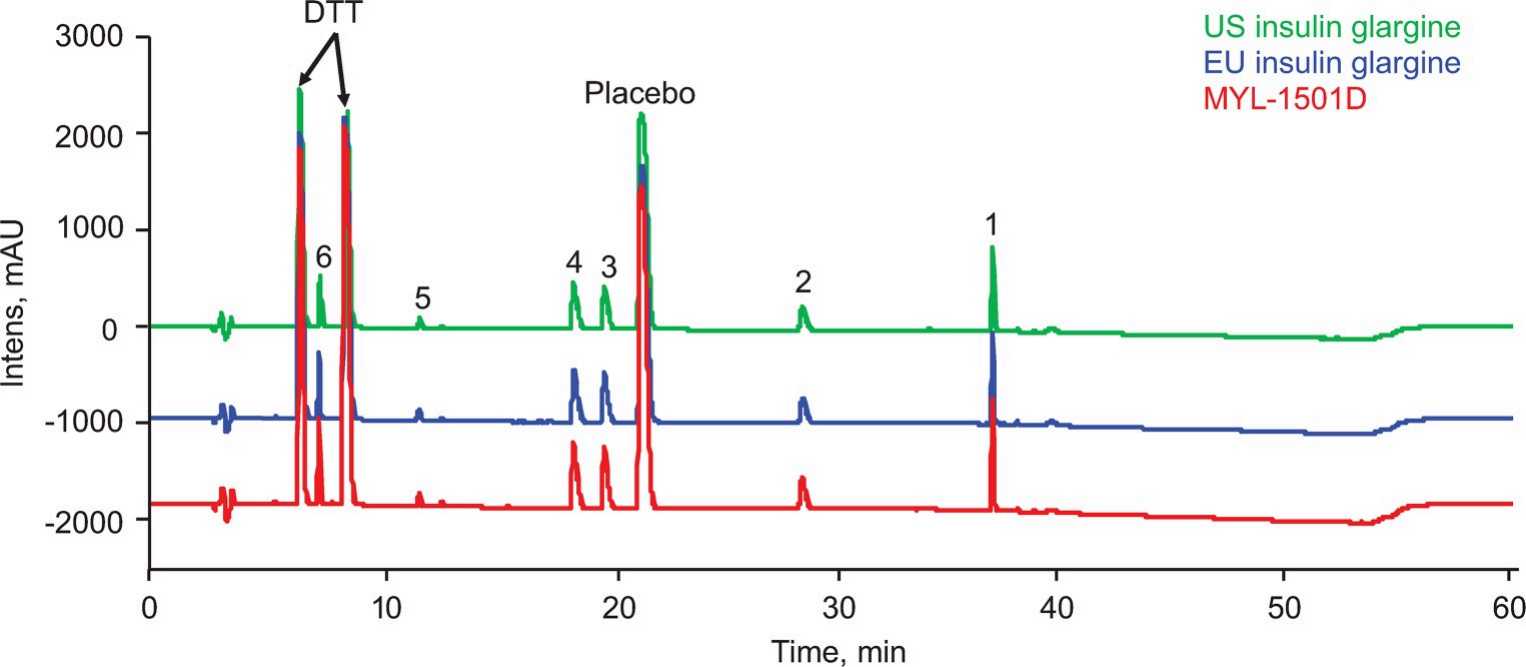
Structural characterization and comparison of MYL-1501D, US- and EU-licensed insulin glargine. Representative overlay of chromatographic peak profile of reduced peptide mass fingerprinting from MYL-1501D and reference products. Fragments 1–6 are described in Table 1. DTT, dithiothreitol.

An overlay of the chromatographic peak profiles of reduced peptide mass fingerprinting for MYL-1501D, US-licensed insulin glargine, and EU-licensed insulin glargine demonstrates a very high similarity among the 3 products, with no new peaks observed for MYL-1501D compared with the reference insulin glargine products (Fig 1). The identified peptide fragments match well to their respective theoretical masses (Table 1).

### Disulfide mapping

In addition to primary structure identification, non-reduced peptide mapping confirmed the presence of 3 disulfide linkages in the insulin glargine preparations: 2 interchain disulfide bonds (A7-B7 and A19-B20) and 1 intrachain disulfide bond (A6-A11). This finding was further corroborated by high-resolution 2D NMR spectroscopy using state-of-the-art correlation experiments such as through-bond (2D TOCSY) and through-space (2D NOESY) spectroscopy (S1 Fig).

### Higher-order structure analysis

Differences in higher-order structure not only provide potential clues about any observed biological and/or immunological differences between proteins and variant forms (ie, proteins with posttranslational modifications) but can also serve as a means for assessing the comparability between a reference product and a biosimilar. Assessment of secondary structural elements (eg, helix, beta sheet, turn, and random coil regions) was deduced from FTIR and far UV-CD spectroscopy, while information on higher-order structures was achieved using multiple orthogonal techniques including near UV-CD, DSC, NMR, and x-ray crystallography.

**Fig 2:**
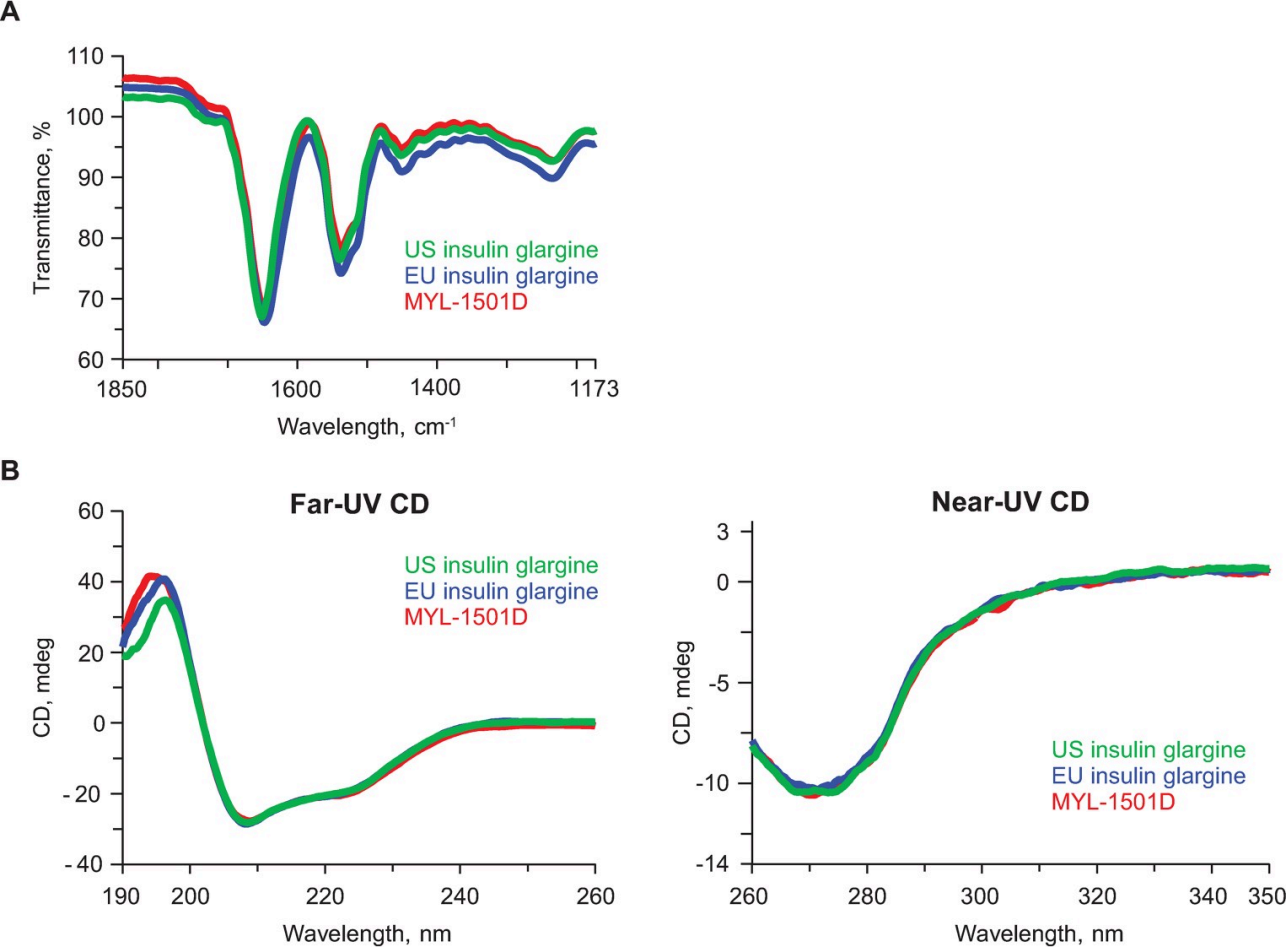
Representative overlay of MYL-1501D, US- and EU-licensed insulin glargine. (A) FTIR Profiles for Secondary Structure of and (B) CD Profiles for Secondary and Tertiary Structures. CD, circular dichroism; FTIR, Fourier-transformed infrared.

FTIR spectra of the 3 drug products were compared by evaluating positions and shapes of the amide I and amide II bands between 1700 and 1500 cm ^-1^. Amide I and amide II bands are primarily caused by carbonyl stretching and NH bending vibrations, respectively. As both carbonyl and NH are involved in hydrogen bonding in protein secondary structures, the position and shape of these bands are highly dependent on the secondary structural elements [25, 26]. FTIR spectra showed high agreement for the 3 insulin glargine products in terms of shape and positions (amide I band at 1647.9 ± 1.5 cm^-1^ and amide II band at 1538.1 ± 1.5 cm^-1^; Fig 2A and Table 1), thus indicating similarity in the secondary structure content across all 3 products. The presence of the amide I band at 1647.9 ± 1.5 cm^-1^ indicates the prominence of alpha helix structures.

Alternatively, far-UV CD also showed characteristic spectra of an alpha helix, with positive maxima at 193 cm^-1^ and negative maxima at 208 and 222 cm^-1^ (Fig 2B, left panel). The ellipticity at each of these wavelengths was highly similar for the 3 drug products, indicating high similarity in the secondary structure content and corroborating the findings from FTIR spectroscopy.

**Fig 3:**
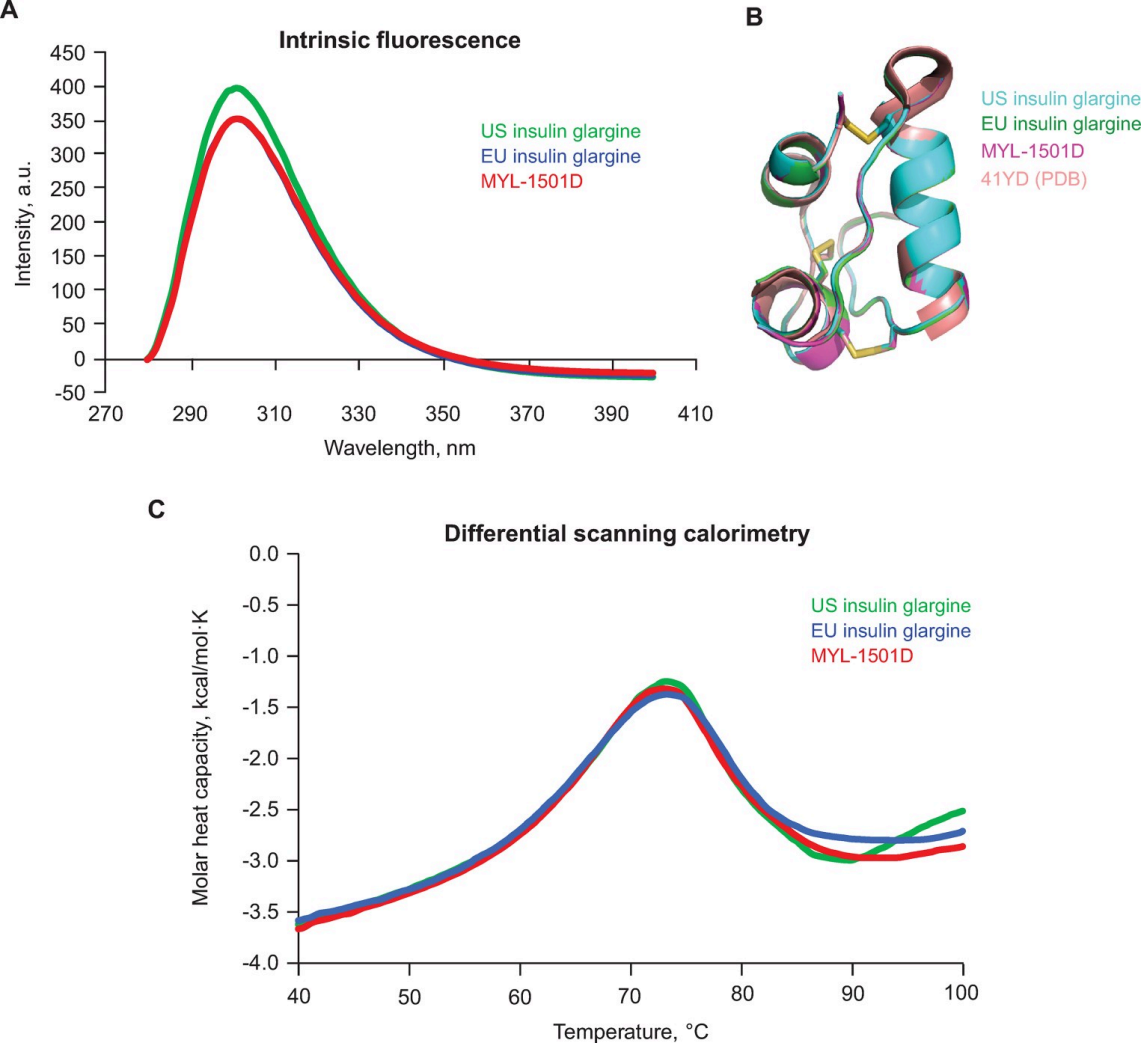
Representative overlay of MYL-1501D, US- and EU-licensed insulin glargine. (A) Intrinsic Fluorescence Spectral Plots (B) Superposition of MYL-1501D X-Ray Structure and Reference Products With Insulin Glargine (4IYD) as the Base. (C) Differential Scanning Calorimetry Profiles of MYL-1501D (Red), US-Licensed Insulin Glargine (Green), and EU-Licensed Insulin Glargine (Blue).

Tertiary structure information on MYL-1501D and reference insulin glargine products was obtained from near UV-CD, intrinsic fluorescence, NMR spectroscopy, and x-ray crystallography. A highly similar near UV-CD (Fig 2B, right panel) and intrinsic fluorescence spectral profile (Fig 3A) for MYL-1501D and the reference products suggest close similarity in the tertiary structures of these products.

High-resolution structural information was extracted from NMR spectroscopy and x-ray crystallography studies. NMR signals are sensitive to their chemical environment, and therefore similar NMR spectra would indicate highly similar solution conformations. NMR experiments that are used to map disulfide bridging (TOCSY, through bond; NOESY, through space) also provided detailed atomic information on proton connectivity across the structure, thus providing high-resolution information on the structure. Highly similar 2D TOCSY and 2D NOESY spectra for the 3 drug products indicate a highly similar 3-dimensional structural arrangement (S1 Fig). This information was further confirmed by x-ray structures, which were obtained at or below a resolution of 2.0 Å for all molecules evaluated (Fig 3B). MYL-1501D and reference products superimpose well with the published structure of insulin glargine from the Protein Data Bank (PDB ID: 4IYD), with an overall root-mean-square deviation value of 0.146 Å (Fig 3B). Close resemblance of the MYL-1501D structure to those of the US- and EU-licensed insulin glargine products confirmed the presence of similar polypeptide folds, disulfide bridging, and structural arrangement.

Differential scanning calorimetry is a stability-indicating tool used to investigate tertiary/quaternary structures. An overlay of representative DSC thermograms for MYL-1501D and US- and EU-licensed insulin glargine products demonstrates a high similarity in unfolding temperatures (T_m_ = 71.5°C ± 1.5°C), indicating similar thermal stability and conformation for the 3 insulin glargine products (Fig 3C).

Altogether, these techniques showed high structural similarity between the MYL-1501D and US- and EU-licensed insulin glargine.

### Content analysis

The concentration of insulin glargine in MYL-1501D and reference products was determined using RP-HPLC. Insulin glargine content in all 3 products was highly similar and observed to be within the range of 95% to 105% (Table 1).

### Purity and impurity analysis

**Fig 4:**
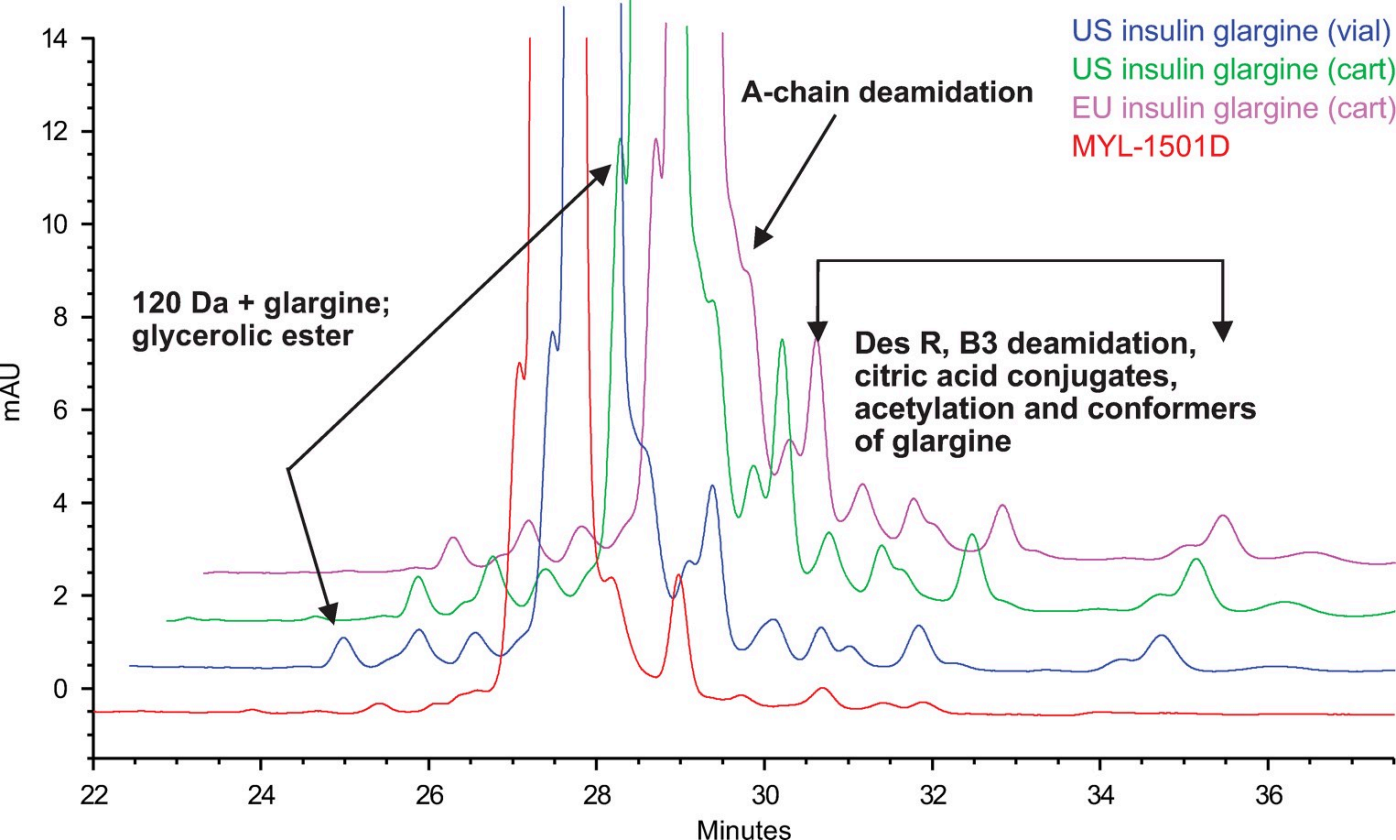
Reverse-phase high-performance liquid chromatography overlay of MYL-1501D, US- and EU-licensed insulin glargine. MYL-1501D (Red), US-Licensed Insulin Glargine (Blue, Vial; Green, Cartridge), and EU-Licensed Insulin Glargine (Magenta).

The purification process for MYL-1501D is designed to remove protein variants that arise from *P pastoris* (the yeast expression system used for production) as well as other process-related impurities generated during manufacturing. Glycan impurities are the most commonly generated protein variants from the yeast expression system and have the potential to be immunogenic. Thus, it is important to characterize all residual protein variants and impurities in the final purified drug product to assess their impact on efficacy and stability. RP-HPLC was used to identify product-related variants generated by deamidation/clipping of the B-chain C-terminal amino acids, mis-cleavage of the insulin glargine precursor by trypsin, or glycosylation (Fig 4). No new peaks were observed for MYL-1501D, and no peaks were detected between 0.82 and 0.98 RRT, which is the range in which mono-, di-, and triglycosylated insulin glargine would have eluted, if present. Product variants for the MYL-1501D lots were within the quality ranges established for US- and EU-licensed insulin glargine (Table 2), and the glycosylated species in the MYL-1501D drug substance were controlled below the quantification limits (0.04%). Considering their potential immunogenicity, these glycosylated impurities have been characterized in detail and have been identified as mono-, di-, and tri-glycosylated variants of drug product. Because of their shorter glycan pattern, the potential for immunogenicity risk is low, and these species do not impact the similarity assessment, and thus, MYL-1501D was considered similar to the reference products.

Size variants such as HMWP species, including aggregates that form because of the association of 2 or more monomers or fragments, were identified by SE-HPLC and orthogonal methods such as multiple angle light scattering detection in tandem with size-exclusion chromatography (SEC-MALS) and AUC. SE-HPLC results demonstrated that the total HMWP content for most of the MYL-1501D, US-licensed insulin glargine, and EU-licensed insulin glargine lots was below the quantification limit (0.05%), and the HMWP content of all tested MYL-1501D lots was within the quality ranges established for the reference products, thus demonstrating high similarity between the 3 products (Table 2). SEC-MALS is used to estimate the molar mass of monomeric and higher oligomeric species. Exponential/Polynomial fitting of results helps determine the predominant molar mass. SEC-MALS analysis of MYL-1501D and reference insulin glargine products demonstrated a similar size range for the monomeric form across all 3 products (Table 2). A single predominant peak of monomer is observed in all samples, with a similar distribution of molar mass. Multimer and aggregate content was too low in all 3 products to provide a measurement of molar mass. These data corroborate the conclusions from UV detection in SE-HPLC.

Sedimentation velocity measured by AUC provides information on protein heterogeneity and the state of association or aggregation. The sedimentation coefficients (*S*) observed for MYL-1501D and US- and EU-licensed insulin glargine were in the range of 1.60 to 1.68 (Table 2), establishing the monodisperse higher-order folded protein structure of insulin glargine and indicating similarity in overall molecular size and shape across all samples.

### Functional characterization of insulin glargine preparations

Studies characterizing functional and biological attributes showed high similarity among MYL-1501D and reference insulin glargine products.

#### Insulin receptor–binding kinetics

**Fig 5:**
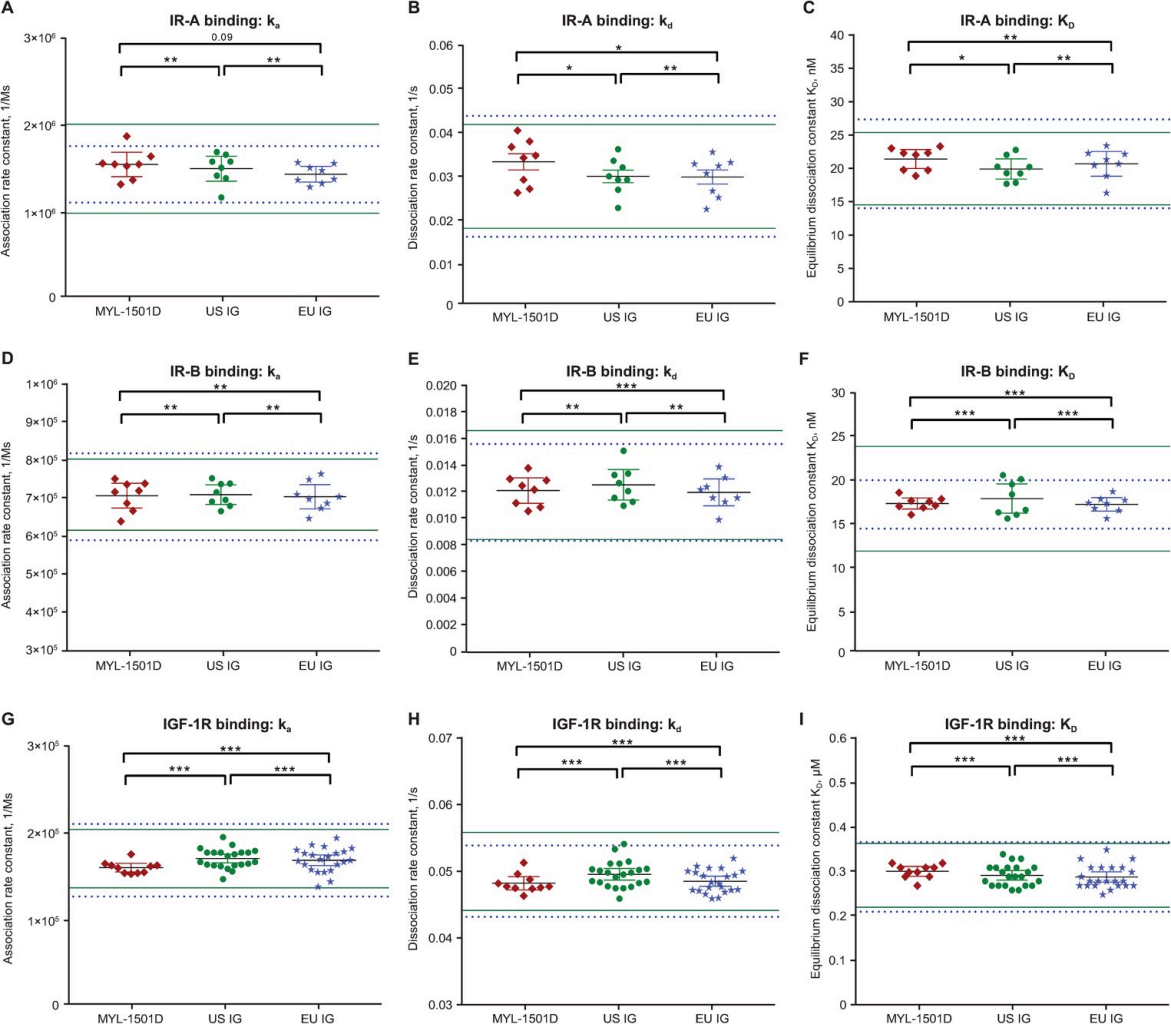
Scatter plot distributions of binding kinetic constants of MYL-1501D, US- and EU-licensed insulin glargine. Binding Kinetic Constants i.e., ka, kd, and K_D_ for (A-C) IR-A (Short-Form), (D-F) IR-B (Long-Form), and (G-I) IGF-1R for MYL-1501D, US-Licensed Insulin Glargine, and EU-Licensed Insulin Glargine. Multiple lots of MYL-1501D, US-, and EU-licensed insulin glargine were analyzed, and data are expressed as mean ± 95% CI. Each dot represents one lot for which data were collected from 3 independent runs, and mean is presented. Mean ± 3 SD range derived from innovator product is presented as solid green line for US-licensed insulin glargine and dotted blue line for EU-licensed insulin glargine. *P<0.05, **P<0.01, and ***P<0.001 in TOST/Equivalence test. IGF-1R, insulin growth factor-1 receptor; IR, insulin receptor; k_a_, association rate constant; k_d_, dissociation rate constant; K_D_, equilibrium dissociation constant; TOST, Two One-Sided T-test.

**Fig 6:**
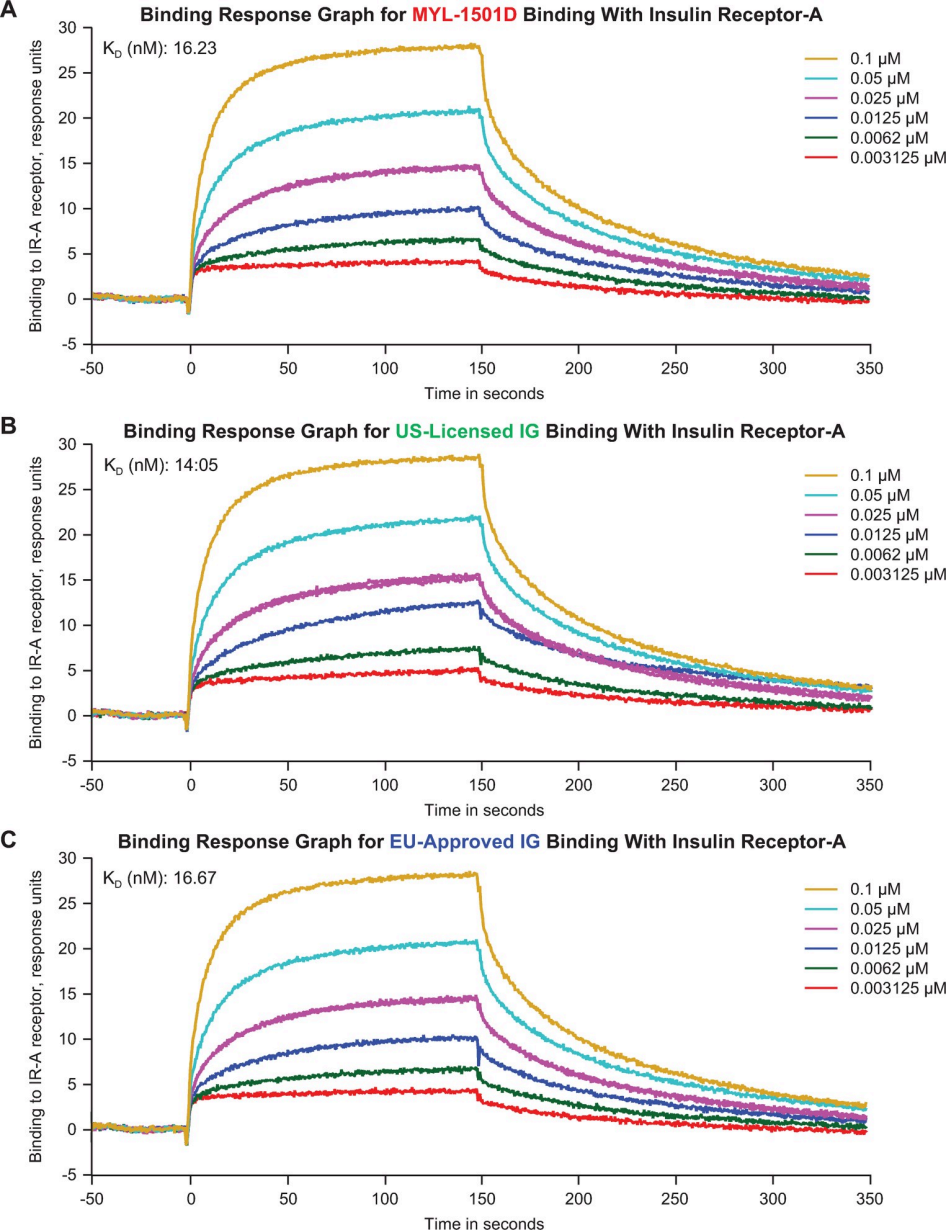
Representative binding response graphs of the insulin receptor-A (short-form) kinetic binding assay. (A) MYL-1501D, (B) US-Licensed Insulin Glargine, and (C) EU-Licensed Insulin Glargine. Each lot was analyzed over 6 concentrations, and kinetic parameters were evaluated by 1:1 interaction model using global fit. Each lot was analyzed 3 independent times, and mean K_D_ value was reported. K_D_, equilibrium dissociation constant.

**Fig 7:**
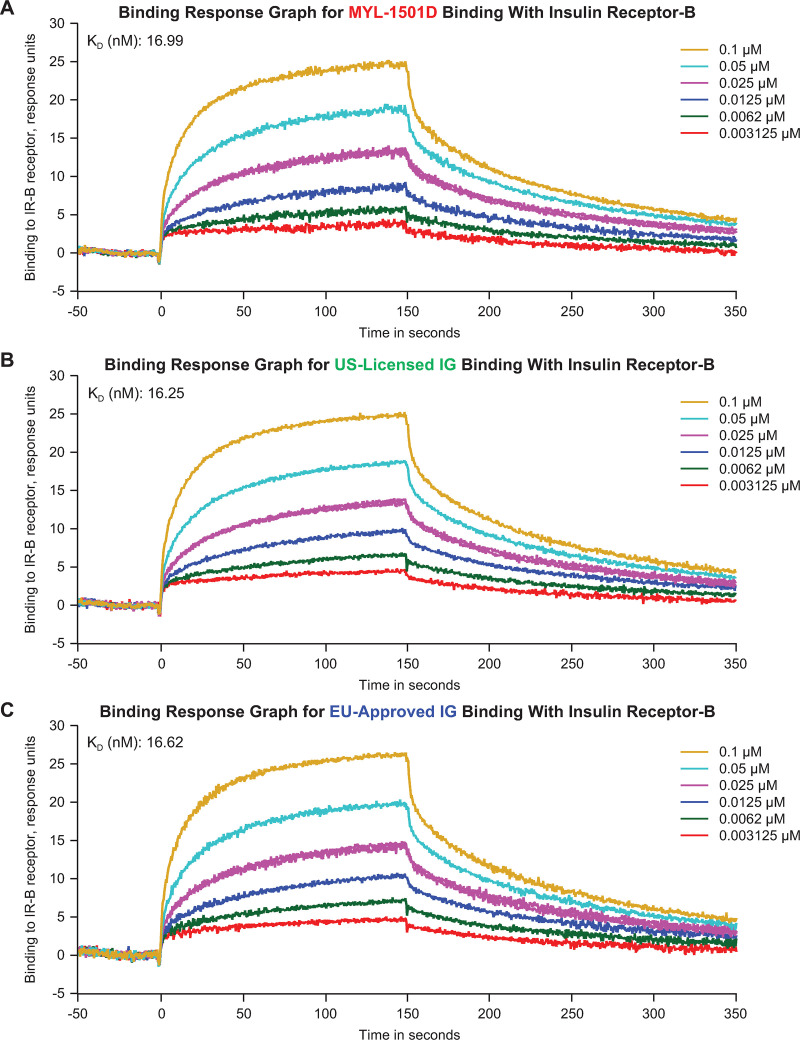
Representative binding response graphs of the insulin receptor-B (long-form) kinetic binding assay. **(**A) MYL-1501D, (B) US-Licensed Insulin Glargine, and (C) EU-Licensed Insulin Glargine. Each lot was analyzed over 6 concentrations, and kinetic parameters were evaluated by 1:1 interaction model using global fit. Each lot was analyzed 3 independent times, and mean K_D_ value was reported. K_D_, equilibrium dissociation constant.

**Fig 8:**
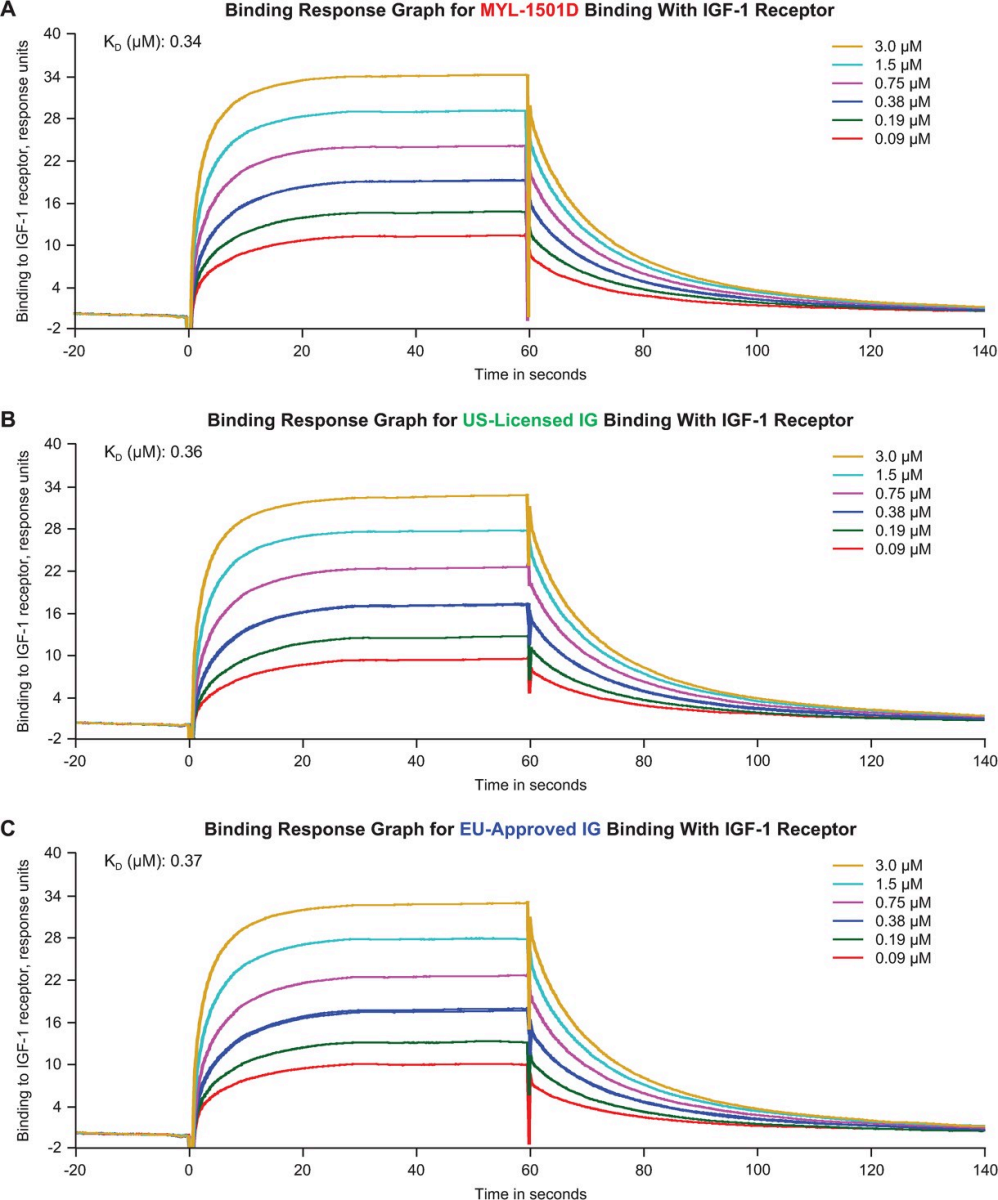
Representative binding response graph of insulin-like growth factor receptor-1 binding kinetics assay. (A) MYL-1501D, (B) US-Licensed Insulin Glargine, and (C) EU-Licensed Insulin Glargine. Each lot was analyzed over 6 concentrations, and kinetic parameters were evaluated by 1:1 interaction model using global fit. Each lot was analyzed 3 independent times, and mean K_D_ value was reported. K_D_, equilibrium dissociation constant.

Insulin receptor–binding kinetics to IR-A, IR-B, and IGF-1R were highly similar according to rates of k_a_, k_d_, and K_D_ (Table 3; Fig 5). Representative sensorgrams are shown for MYL-1501D and reference products for the IR-A (Fig 6), IR-B (Fig 7), and IGF-1R (Fig 8) binding kinetics assays. The *P* value as calculated by the TOST analysis for all groups and assays confirmed equivalence except between MYL-1501D and EU-licensed insulin glargine for associate rate constant, ie, k_a_ for IR-A binding kinetics, where the *P* value was marginally higher than 0.05 (ie, 0.09). However, the K_D_ value, which represents the equilibrium dissociation constant (k_d_/k_a_), demonstrates that these slight differences do not impact overall K_D_ values for IR-A kinetics and hence overall binding was considered comparable for all groups and samples.

**Table 3:** Insulin receptor IR-A, IR-B and IGF-1R, binding kinetics of MYL-1501D, US- and EU-licensed insulin glargine.

Receptor	Binding kinetic parameters	Minimum–maximum observed range (number of lots tested)		
		MYL-1501D	EU reference product	US reference product
IGF-1R	k_a_ (1/Ms × 10^5^)	1.53–1.76 (10)	1.38–1.95 (22)	1.47–1.96 (22)
	k_d_ (1/s)	0.046–0.051 (10)	0.048–0.052 (22)	0.046–0.054 (22)
	K_D_ (μM)	0.27–0.32 (10)	0.25–0.35 (22)	0.26–0.34 (22)
IR-B (long-form)	k_a_ (1/Ms × 10^5^)	6.41–7.54 (8)	6.49–7.67 (8)	6.68–7.55 (8)
	k_d_ (1/s)	0.011–0.014 (8)	0.010–0.013 (8)	0.011–0.015 (8)
	K_D_ (nM)	15.81–18.37 (8)	15.37–18.49 (8)	15.36–20.40 (8)
IR-A (short-form)	k_a_ (1/Ms × 10^6^)	1.33–1.88 (8)	1.30–1.58 (8)	1.18–1.70 (8)
	k_d_ (1/s)	0.026–0.041 (8)	0.022–0.036 (8)	0.023–0.036 (8)
	K_D_ (nM)	18.80–23.29 (8)	16.23–23.39 (8)	17.62–22.75 (8)

Summary of minimum and maximum observed binding kinetic parameters (association rate constant [ka], dissociation rate constant [kd], and equilibrium dissociation constant [KD]) for multiple lots of MYL-1501D and EU and US reference products analyzed using 3 major receptors (IGF-1R, IR-A, and IR-B). The number of lots analyzed for each product is presented in parentheses.

IGF-1R, insulin growth factor-1 receptor; IR, insulin receptor; k_a_, association rate constant; k_d_, dissociation rate constant; K_D_, equilibrium dissociation constant.

#### Insulin receptor phosphorylation

**Fig 9:**
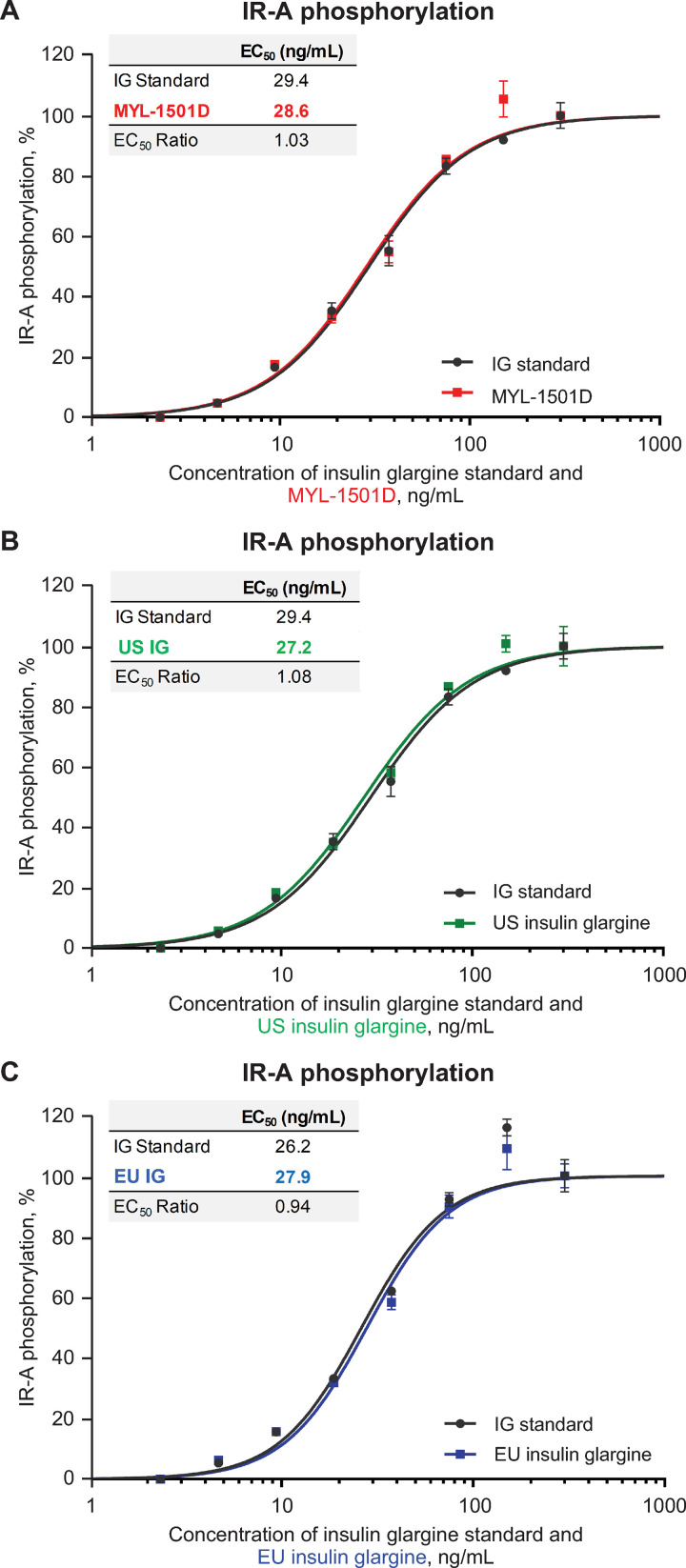
Representative dose-response curves of insulin receptor-A (short-form) phosphorylation assay. Recombinant CHO-K1 Cells Expressing Insulin Receptor-A were used in the assay. (A) MYL-1501D, (B) US-Licensed Insulin Glargine, and (C) EU-Licensed Insulin Glargine. All samples were analyzed against a common insulin glargine reference standard (IG standard) to normalize against day-to-day variability. Each lot was analyzed using 3 independent dilution preparations on an assay plate. Mean ± SEM for the % phosphorylation response is plotted (y-axis) against the concentration (x-axis). Four parameter logistic curve fit was applied to evaluate EC_50_. Each sample was analyzed on 3 independent assay plates, and average is reported. CHO, Chinese hamster ovary; EC_50_, half maximal effective concentration.

**Fig 10:**
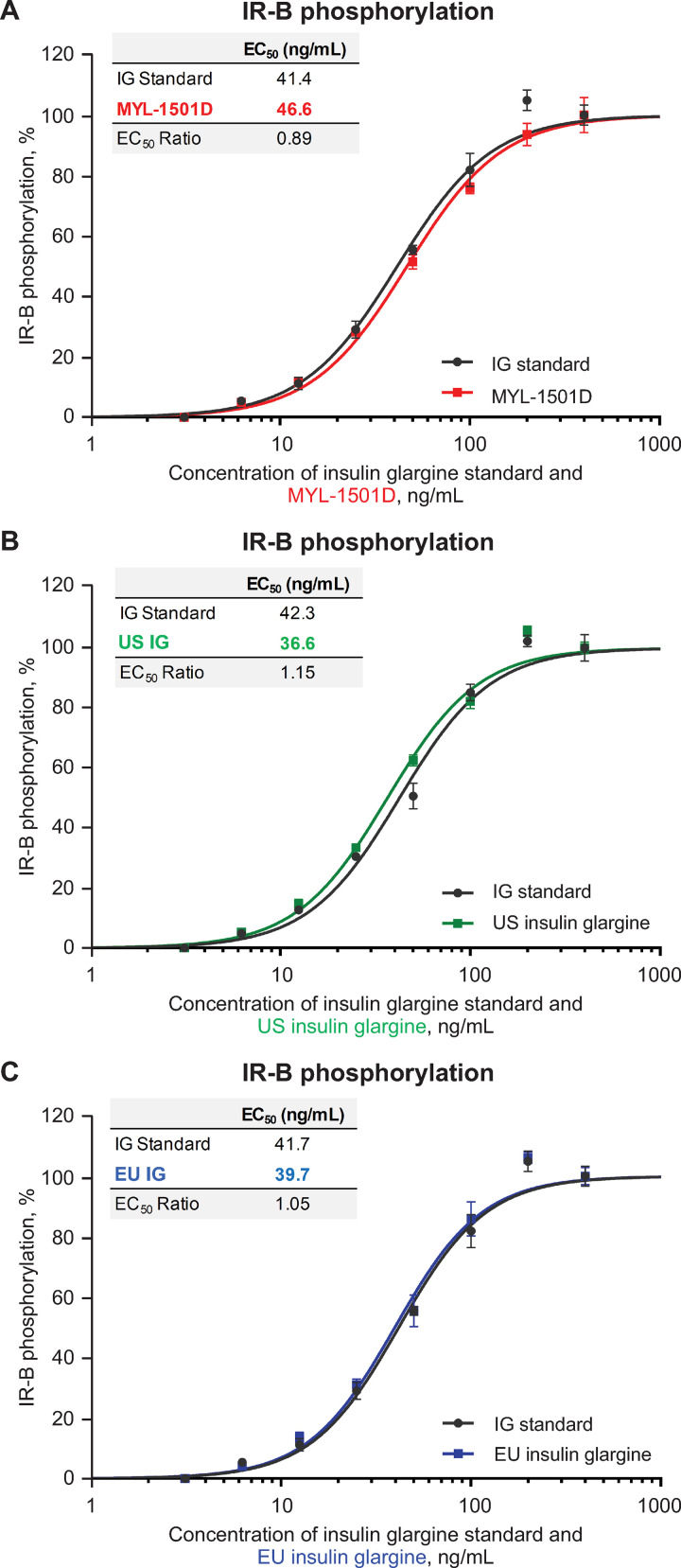
Representative dose-response curves of insulin receptor-B (long-form) phosphorylation assay. Recombinant CHO-K1 Cells Expressing Insulin Receptor-B were used in the assay. (A) MYL-1501D, (B) US-Licensed Insulin Glargine, and (C) EU-Licensed Insulin Glargine. All samples were analyzed against a common insulin glargine reference standard (IG standard) to normalize against day-to-day variability. Each lot was analyzed using 3 independent dilution preparations on an assay plate. Mean ± SEM for the % phosphorylation response is plotted (y-axis) against the concentration (x-axis). Four parameter logistic curve fit was applied to evaluate EC_50_. Each sample was analyzed on 3 independent assay plates, and average is reported. CHO, Chinese hamster ovary; EC_50_, half maximal effective concentration.

**Fig 11:**
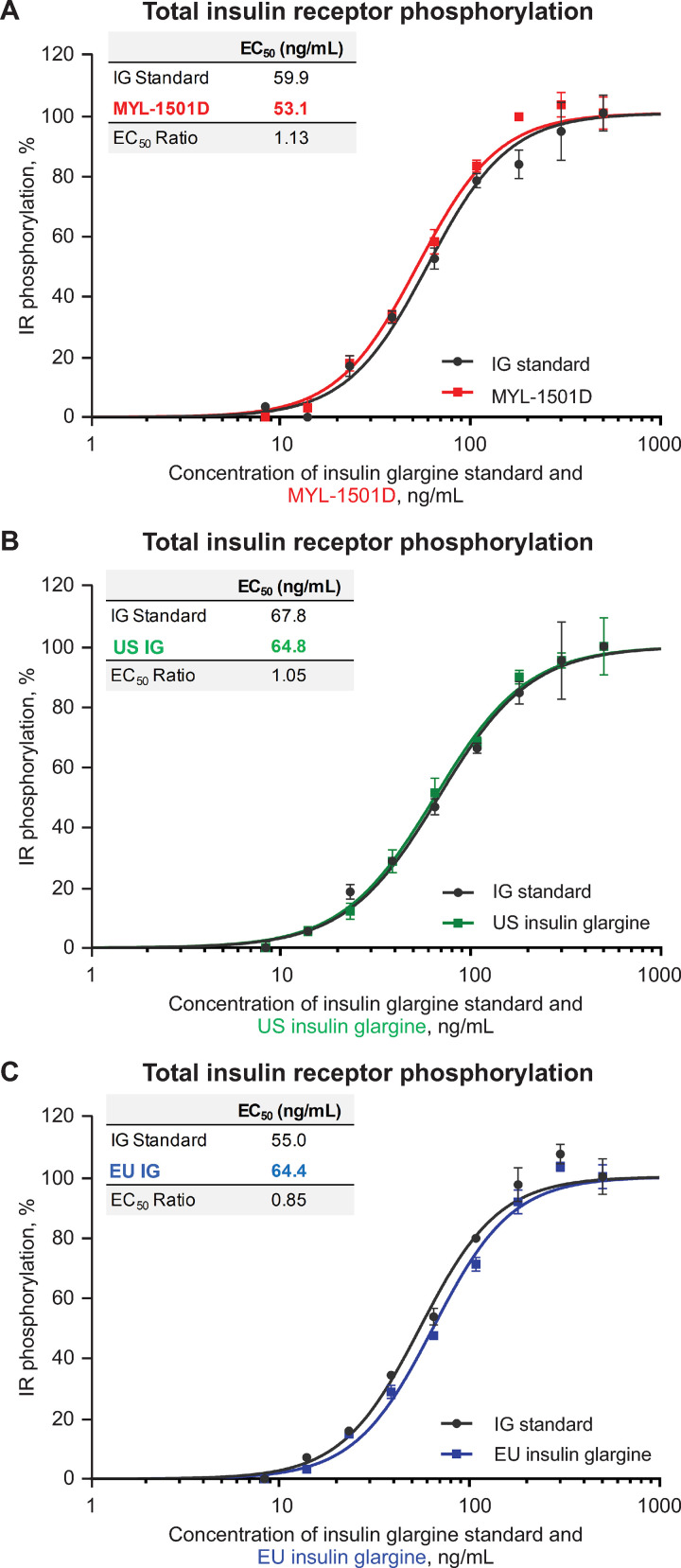
Representative dose-response curves of total insulin receptor phosphorylation assay in HepG2 cells. (A) MYL-1501D, (B) US-Licensed Insulin Glargine, and (C) EU-Licensed Insulin Glargine. All samples were analyzed against a common insulin glargine reference standard (IG standard) to normalize against day-to-day variability. Each lot was analyzed using 3 independent dilution preparations on an assay plate. Mean ± SEM for the % phosphorylation response is plotted (y-axis) against the concentration (x-axis). Four parameter logistic curve fit was applied to evaluate EC_50_. Each sample was analyzed on 3 independent assay plates, and average is reported. CHO, Chinese hamster ovary; EC_50_, half maximal effective concentration.

**Fig 12:**
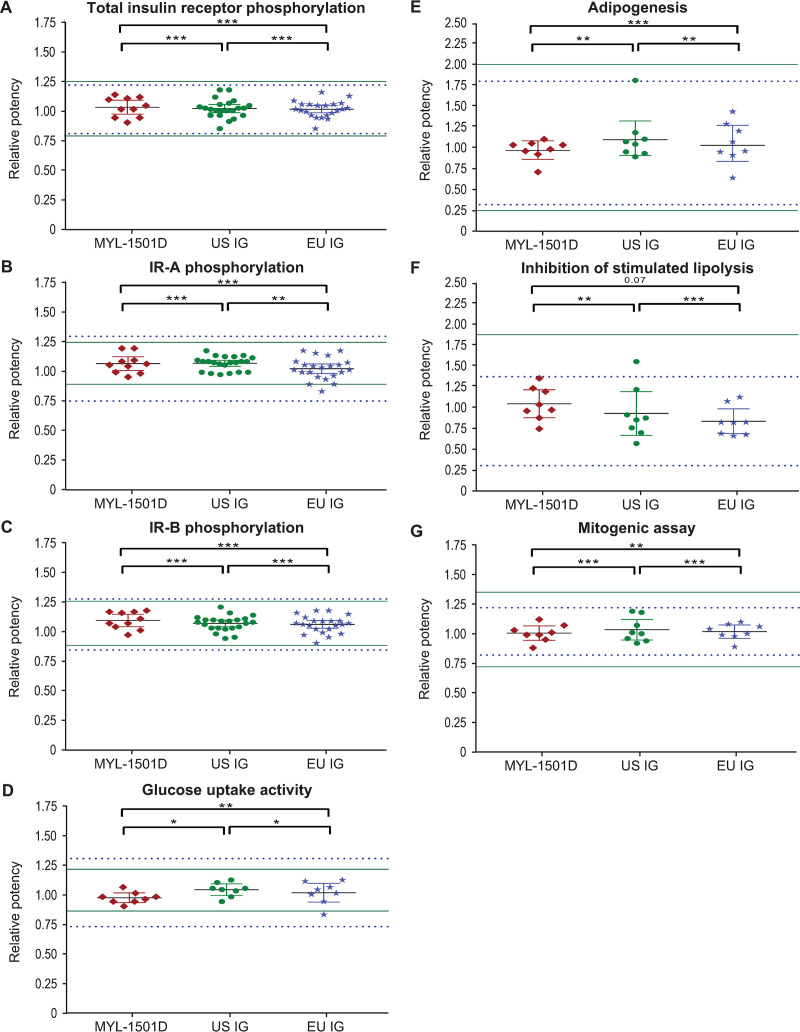
Scatter plot distribution of average relative potency of MYL-1501D, US- and EU-licensed insulin glargine. (A) IR Phosphorylation in HepG2 Cells, (B) IR-A Phosphorylation in CHO-K1 Cells Expressing IR-A, (C) IR-B Phosphorylation in CHO-K1 Cells Expressing IR-B, (D) Glucose Uptake Activity in 3T3-L1 Cells, (E) Adipogenicity Activity in 3T3-L1 Cells, (F) Inhibition of Stimulated Lipolysis Activity in 3T3-L1 Cells, and (G) Mitogenic Activity by Saos-2 Cells. Multiple lots of MYL-1501D, US-, and EU-licensed insulin glargine were analyzed, and data are expressed as mean ± 95% CI. Each dot represents one lot for which data were collected from 3 independent runs, and mean is presented. Mean ± 3 SD range derived from innovator product is presented as solid green line for US-licensed insulin glargine and dotted blue line for EU-licensed insulin glargine. *P<0.05, **P<0.01, and ***P<0.001 in TOST/Equivalence test. CHO, Chinese hamster ovary; GOPOD, glucose oxidase/peroxidase; IR, insulin receptor; Saos-2, sarcoma osteogenic cell; TOST, Two One-Sided T-test.

Representative dose-response curves for MYL-1501D and US- and EU-licensed reference products are shown for the IR-A (Figs 9 and S2), IR-B (Figs 10 and S3), and total insulin receptor (Figs 11 and S4) phosphorylation assays. All phosphorylation studies demonstrated highly similar relative potency for MYL-1501D and reference products across multiple batches (Fig 12A).

#### Metabolic and mitogenic activity

**Fig 13:**
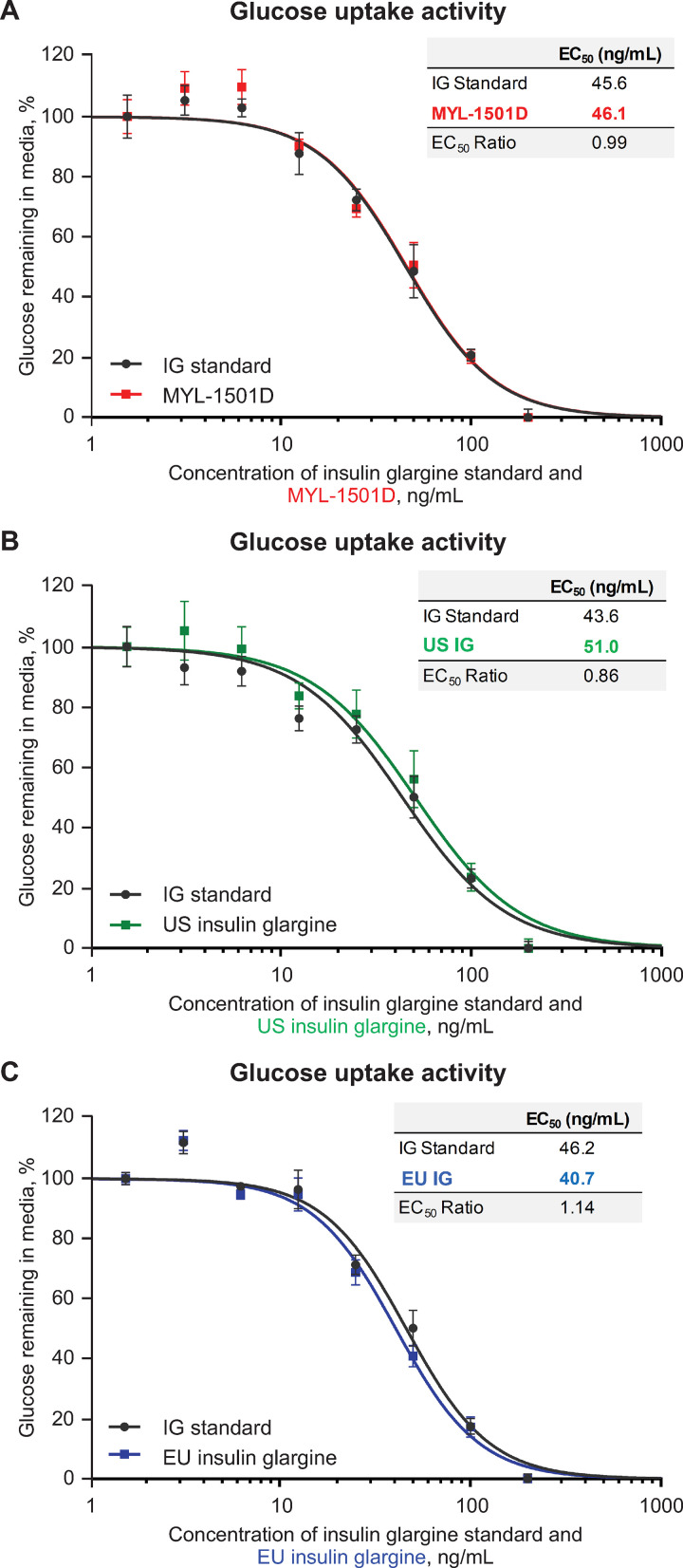
Representative dose-response curves of enzymatic glucose uptake assay in 3T3-L1 cells. (A) MYL-1501D, (B) US-Licensed Insulin Glargine, and (C) EU-Licensed Insulin Glargine. All samples were analyzed against a common insulin glargine reference standard (IG standard) to normalize against day-to-day variability. Each lot was analyzed using 3 independent dilution preparations on an assay plate. Mean ± SEM for the % glucose remaining in the media is plotted (y-axis) against the concentration (x-axis). Four parameter logistic curve fit was applied to evaluate EC_50_. Each sample was analyzed on 3 independent assay plates, and average is reported. EC_50_, half maximal effective concentration.

**Fig 14:**
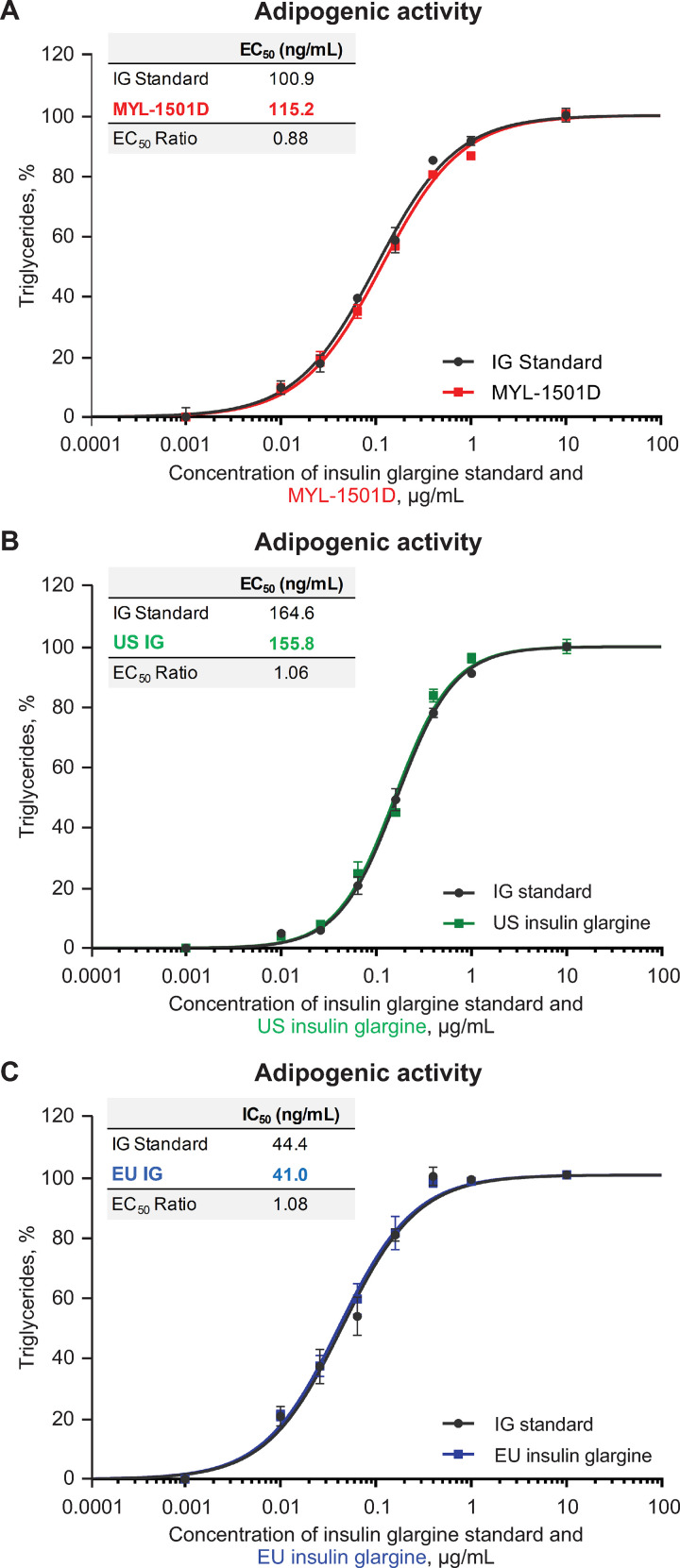
Representative dose-response curves of adipogenesis assay in 3T3-L1 cells. (A) MYL-1501D, (B) US-Licensed Insulin Glargine, and (C) EU-Licensed Insulin Glargine. All samples were analyzed against a common insulin glargine reference standard (IG standard) to normalize against day-to-day variability. Each lot was analyzed using 3 independent dilution preparations on an assay plate. Mean ± SEM for the % triglycerides measured is plotted (y-axis) against the concentration (x-axis). Four parameter logistic curve fit was applied to evaluate EC_50_. Each sample was analyzed on 3 independent assay plates, and average is reported. EC_50_, half maximal effective concentration.

**Fig 15:**
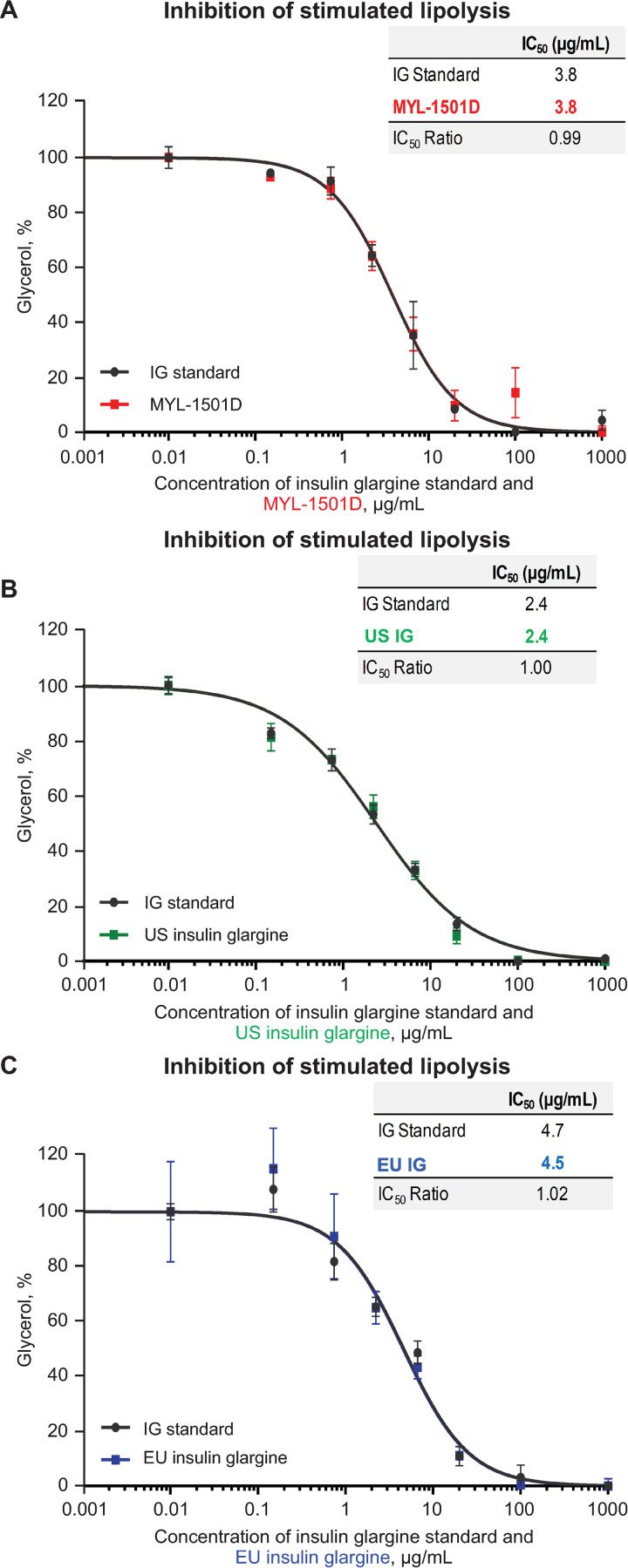
Representative dose-response curves graph of inhibition of stimulated lipolysis assay in 3T3-L1 cells. (A) MYL-1501D, (B) US-Licensed Insulin Glargine, and (C) EU-Licensed Insulin Glargine. All samples were analyzed against a common insulin glargine reference standard (IG standard) to normalize against day-to-day variability. Each lot was analyzed using 3 independent dilution preparations on an assay plate. Mean ± SEM for the % glycerol measured is plotted (y-axis) against the concentration (x-axis). Four parameter logistic curve fit was applied to evaluate EC_50_. Each sample was analyzed on 3 independent assay plates, and average is reported. EC_50_, half maximal effective concentration.

**Fig 16:**
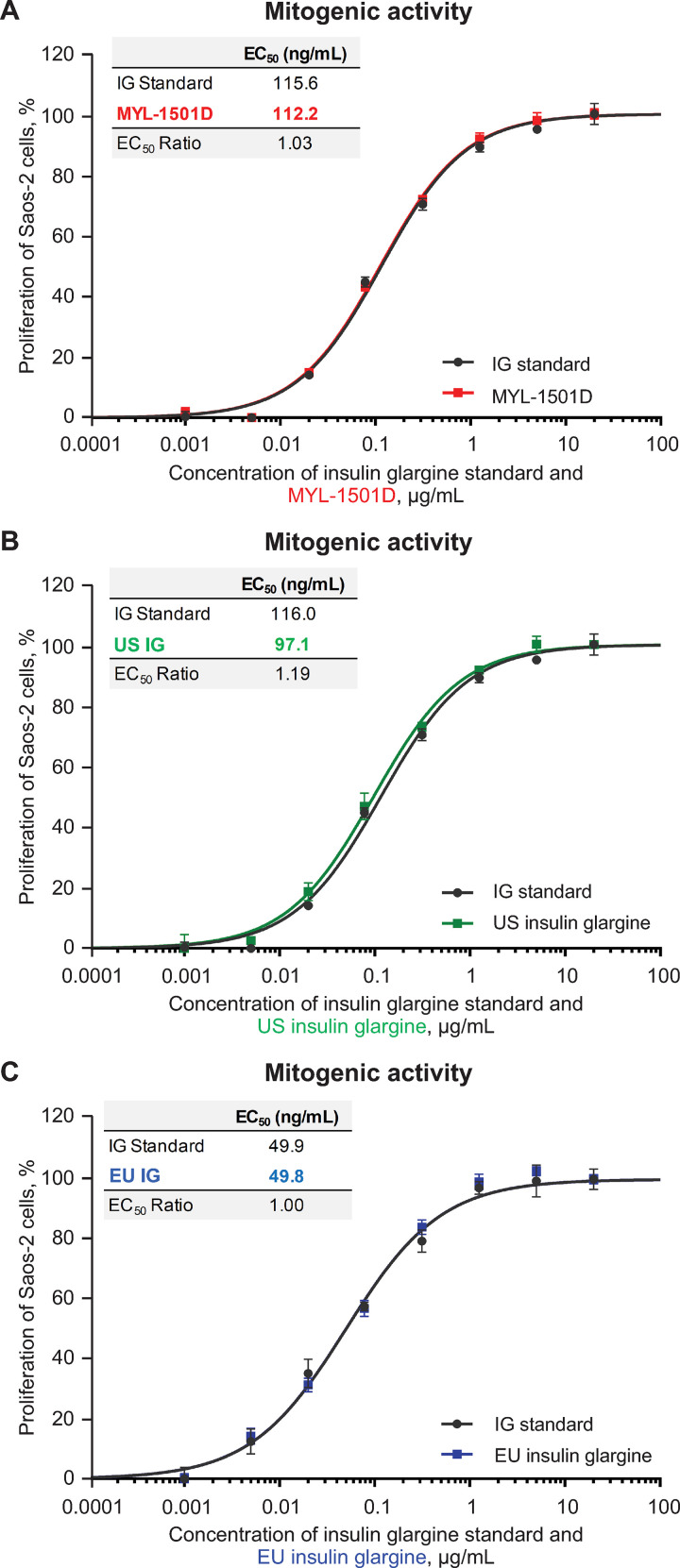
Representative dose-response curves of mitogenic assay in Saos-2 cells. (A) MYL-1501D, (B) US-Licensed Insulin Glargine, and (C) EU-Licensed Insulin Glargine. All samples were analyzed against a common insulin glargine reference standard (IG standard) to normalize against day-to-day variability. Each lot was analyzed using 3 independent dilution preparations on an assay plate. Mean ± SEM for the % proliferation is plotted (y-axis) against the concentration (x-axis). Four parameter logistic curve fit was applied to evaluate EC_50_. Each sample was analyzed on 3 independent assay plates, and average is reported. EC_50_, half maximal effective concentration; Saos-2, sarcoma osteogenic cell.

**Fig 17:**
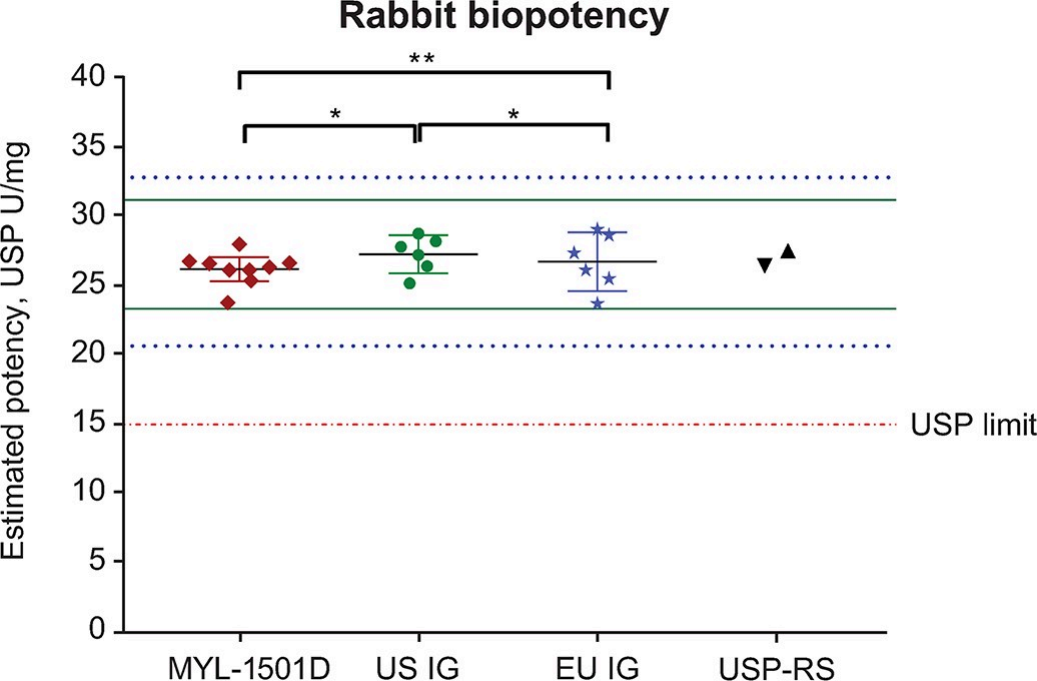
Scatter plot distribution of *in-vivo* rabbit bioassay activity for MYL-1501D, US-, and EU-licensed insulin glargine. Nine lots of MYL-1501D and 6 lots each of US- and EU-licensed insulin glargine were analyzed, and data are expressed as mean ± 95% CI. Mean ± 3 SD range derived from innovator product is presented as solid green line for US-licensed insulin glargine and dotted blue line for EU-licensed insulin glargine. *P<0.05, **P<0.01, and ***P<0.001 in TOST/Equivalence test. Potency of the 2 USP reference standards (USP-RS) used in the study are presented as black symbols (▲ USP human insulin standard lot JOJ250, ▼ USP insulin glargine standard lot F009M0). Limit of the biopotency value, ie, ≥15 USP units/mg, as per USP <121> is presented as red dotted line. TOST, Two One-Sided T-test; USP, US Pharmacopeia.

Mean relative metabolic potency measures of MYL-1501D according to glucose uptake (Figs 12B, 13 and S5), adipogenesis activity (Figs 12B and 14), and inhibition of stimulated lipolysis (Figs 12B and 15) were all within quality ranges of the reference products and considered highly similar. The *P* value as calculated by the TOST analysis for all groups and assays confirmed equivalence except between MYL-1501D and EU-licensed insulin glargine for inhibition of stimulated lipolysis where the *P* value was marginally higher than 0.05 (ie, 0.07). This may be due to high variability of the assay. However, as all MYL-1501D lots were within mean ± 3 SD range of EU-licensed insulin glargine, the groups were considered comparable. Relative mitogenic potency according to proliferation of Saos-2 cells was also found to be highly similar across lots of all the insulin glargine preparations (Figs 12C, 16 and S6). According to USP <121> standards, relative potency, measured by *in vivo* blood glucose assay, was similar for MYL-1501D, the EU-licensed preparation, and US-licensed insulin glargine (Fig 17).

## Discussion

The panel of structural and functional studies conducted to evaluate the comparability of MYL-1501D to US- and EU-licensed preparations of insulin glargine demonstrated high similarity among the follow-on biologic and reference products. The critical quality attributes of MYL-1501D, which were defined through a formal established process set forth by the ICH, were evaluated using state-of-the-art scientific methods and standards.

All physicochemical and functional methods yielded comparable spectral or chromatographic profiles among the evaluated MYL-1501D and US- and EU-licensed insulin glargine lots. Peptide mass fingerprinting analyses showed that the primary structural sequence of MYL-1501D was identical to those of the reference insulin glargine products. Secondary and tertiary protein structures, which carry information on higher-order structure, were also highly similar among MYL-1501D and reference products as evaluated using multiple orthogonal techniques. Additionally, measures of molecular mass, protein content, purity and variants, metabolic and mitogenic activity, and in vivo potency all revealed high similarity of MYL-1501D to reference product preparations. The extensive characterization discussed in this report provides a strong basis for the demonstration of biosimilarity of MYL-1501D with reference products. Additionally, clinical efficacy and safety of MYL-1501D vs insulin glargine in patients with type 1 or type 2 diabetes have been confirmed in the INSTRIDE 1 [12] and INSTRIDE 2 [13] studies, respectively, and were used to support approval of MYL-1501D in the United States and European Union. Based on the totality of evidence, MYL-1501D has been demonstrated to be similar/comparable to US- and EU-licensed insulin glargine products according to the regulatory expectations of the United States and European Union.

The *P pastoris* expression system was selected for the development of MYL-1501D based on its established merits [18, 20, 27], even though reference insulin glargine is based on an *E coli* system. Yeast-based expression systems rapidly evolved after historical discoveries led to analogue insulin production in the *E coli* expression system [20, 28]. The purification process for MYL-1501D reduces glycosylated impurities to below quantifiable limits, and repeat-dose toxicology (unpublished data) and clinical studies [12, 13] with MYL-1501D drug product demonstrated similarity of MYL-1501D and insulin glargine, indicating a negligible impact of the *P pastoris* expression system on the insulin glargine drug product.

Biosimilar and follow-on biologic insulin products are equally safe, and due to their low cost, are expected to offer additional choices and access to clinicians and a wider group of patients evaluating insulin therapy options for DM [29]. Regulatory pathways and approvals have varied worldwide, but regulators and medical specialty societies have consistently emphasized the importance of similarity and safety among follow-on biologic insulins [30, 31]. A recent systematic review of data published through January 2018 illustrated similar clinical efficacy, safety, and immunogenicity among follow-on biologics and reference insulin products [32]. This report offers further evidence of high structural and functional similarity between MYL-1501D insulin glargine and the US- and EU-licensed reference products, which have been available to patients with DM since 2000 [3].

## Supporting information

S1 Fig Extracted NMR spectra of the amide region. 2D [1H, 1H] TOCSY (Left) and 2D [1H, 1H] NOESY (Right) of (A) MYL-1501D, (B) US-Licensed Insulin Glargine, and (C) EU-Licensed Insulin Glargine. Vertical dotted lines indicate spectral assignments for cysteine molecules at positions A6, A7, A11, A20, B7, and B19. Horizontal dotted lines show one nuclear Overhauser effect resulting from the disulfide linkage, indicated as hyphenated residue numbers. NMR, nuclear magnetic resonance.
(TIF)

S2 Fig Representative Parallel-Line Assessment (PLA) curves of insulin receptor-A (short-form) phosphorylation assay. (A) MYL-1501D, (B) US-Licensed Insulin Glargine, and (C) EU-Licensed Insulin Glargine.
(TIF)

S3 Fig Representative Parallel-Line Assessment (PLA) curves of insulin receptor-B (long-form) phosphorylation assay. (A) MYL-1501D, (B) US-Licensed Insulin Glargine, and (C) EU-Licensed Insulin Glargine.
(TIF)

S4 Fig Representative Parallel-Line Assessment (PLA) curves of total insulin receptor phosphorylation assay in HepG2 cells. (A) MYL-1501D, (B) US-Licensed Insulin Glargine, and (C) EU-Licensed Insulin Glargine.
(TIF)

S5 Fig Representative Parallel-Line Assessment (PLA) curves of enzymatic glucose Uptake assay. (A) MYL-1501D, (B) US-Licensed Insulin Glargine, and (C) EU-Licensed Insulin Glargine. PLA, parallel-line assessment.
(TIF)

S6 Fig Representative Parallel-Line Assessment (PLA) curves of mitogenic assay. (A) MYL-1501D, (B) US-Licensed Insulin Glargine, and (C) EU-Licensed Insulin Glargine.
(TIF)

S1 Table Risk ranking of insulin glargine quality attributes. (PDF)
