# Supplementary figures and images for: Physicochemical and functional characterization of MYL-1501D, a proposed biosimilar to insulin glargine
Source: PLoS One. 2021 Jun 16;16(6):e0253168. doi: 10.1371/journal.pone.0253168 (PMC8208551; doi:10.1371/journal.pone.0253168)

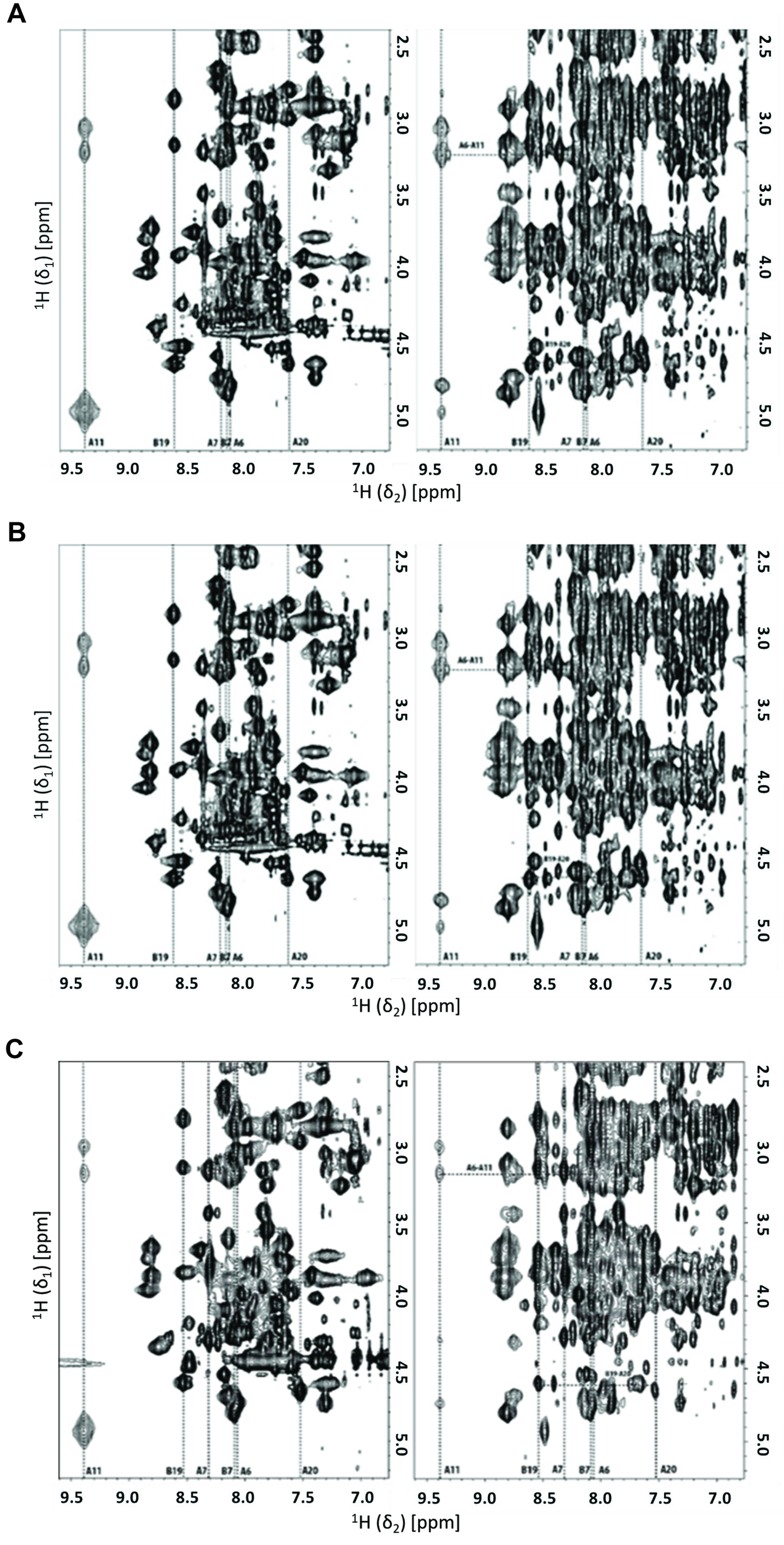

Supplement: S1 Fig — 2D [1H, 1H] TOCSY (Left) and 2D [1H, 1H] NOESY (Right) of (A) MYL-1501D, (B) US-Licensed Insulin Glargine, and (C) EU-Licensed Insulin Glargine. Vertical dotted lines indicate spectral assignments for cysteine molecules at positions A6, A7, A11, A20, B7, and B19. Horizontal dotted lines show one nuclear Overhauser effect resulting from the disulfide linkage, indicated as hyphenated residue numbers. NMR, nuclear magnetic resonance. (TIF) [file pone.0253168.s001.tif]

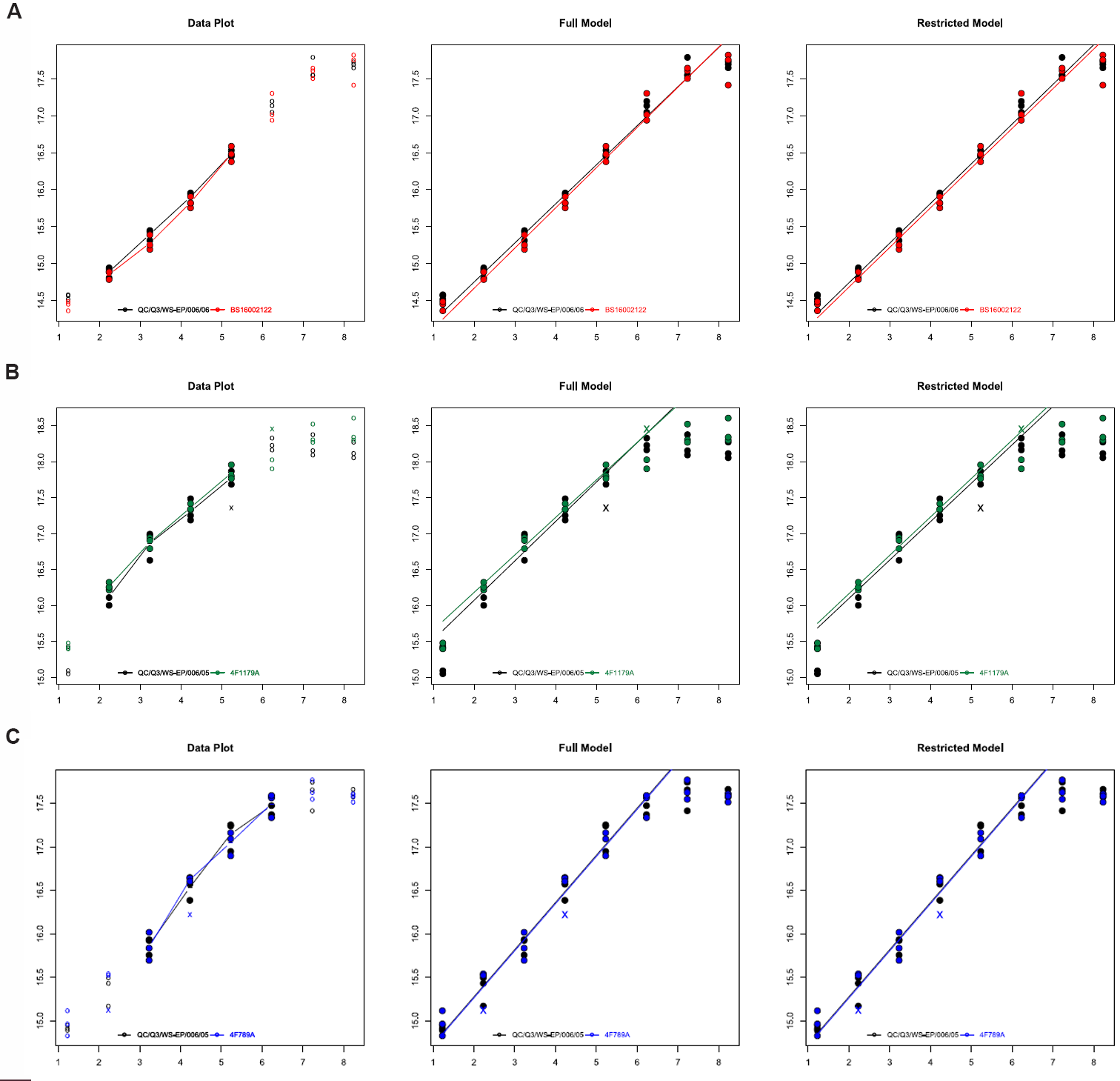

Supplement: S2 Fig — (A) MYL-1501D, (B) US-Licensed Insulin Glargine, and (C) EU-Licensed Insulin Glargine. (TIF) [file pone.0253168.s002.tif]

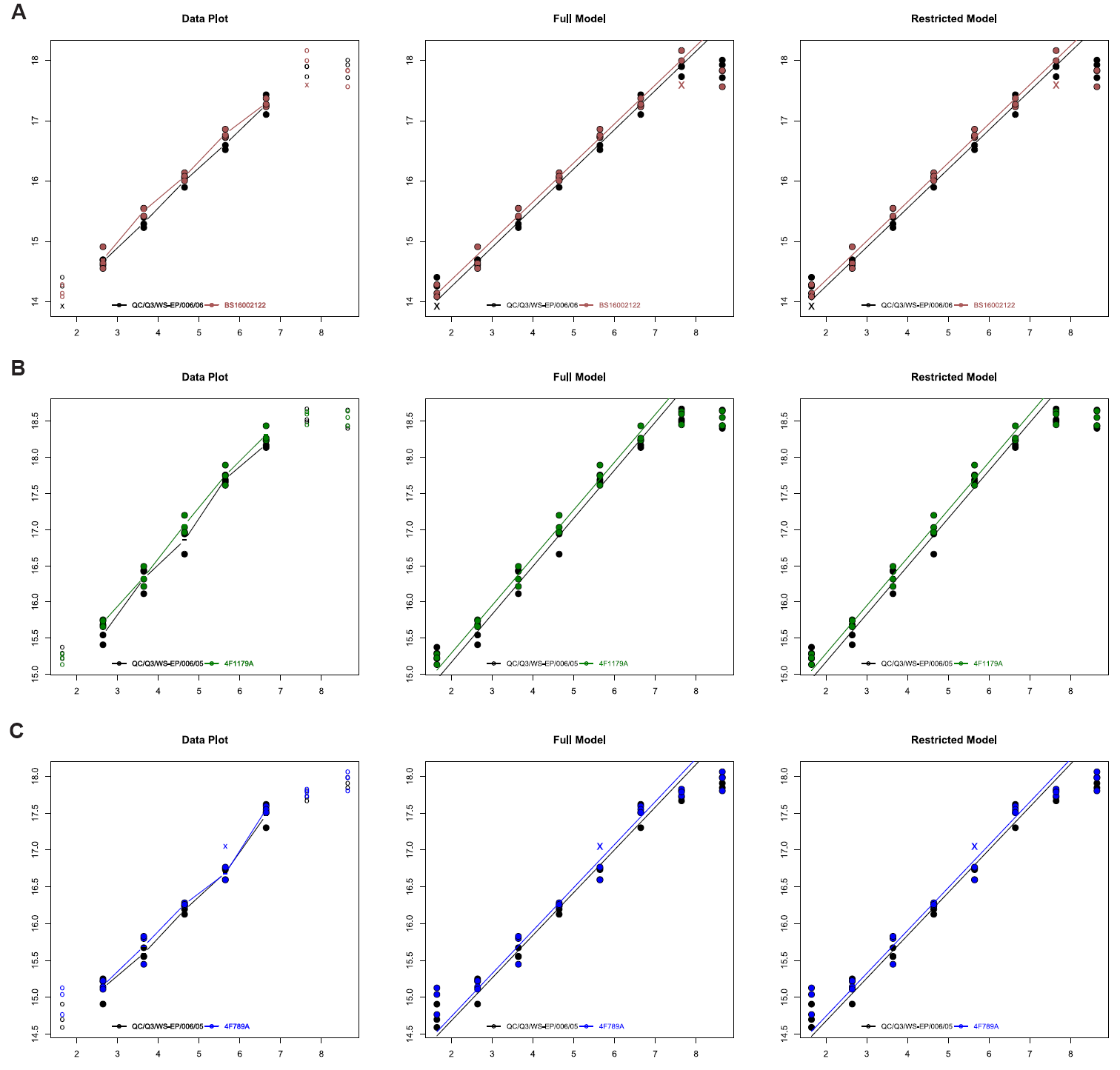

Supplement: S3 Fig — (A) MYL-1501D, (B) US-Licensed Insulin Glargine, and (C) EU-Licensed Insulin Glargine. (TIF) [file pone.0253168.s003.tif]

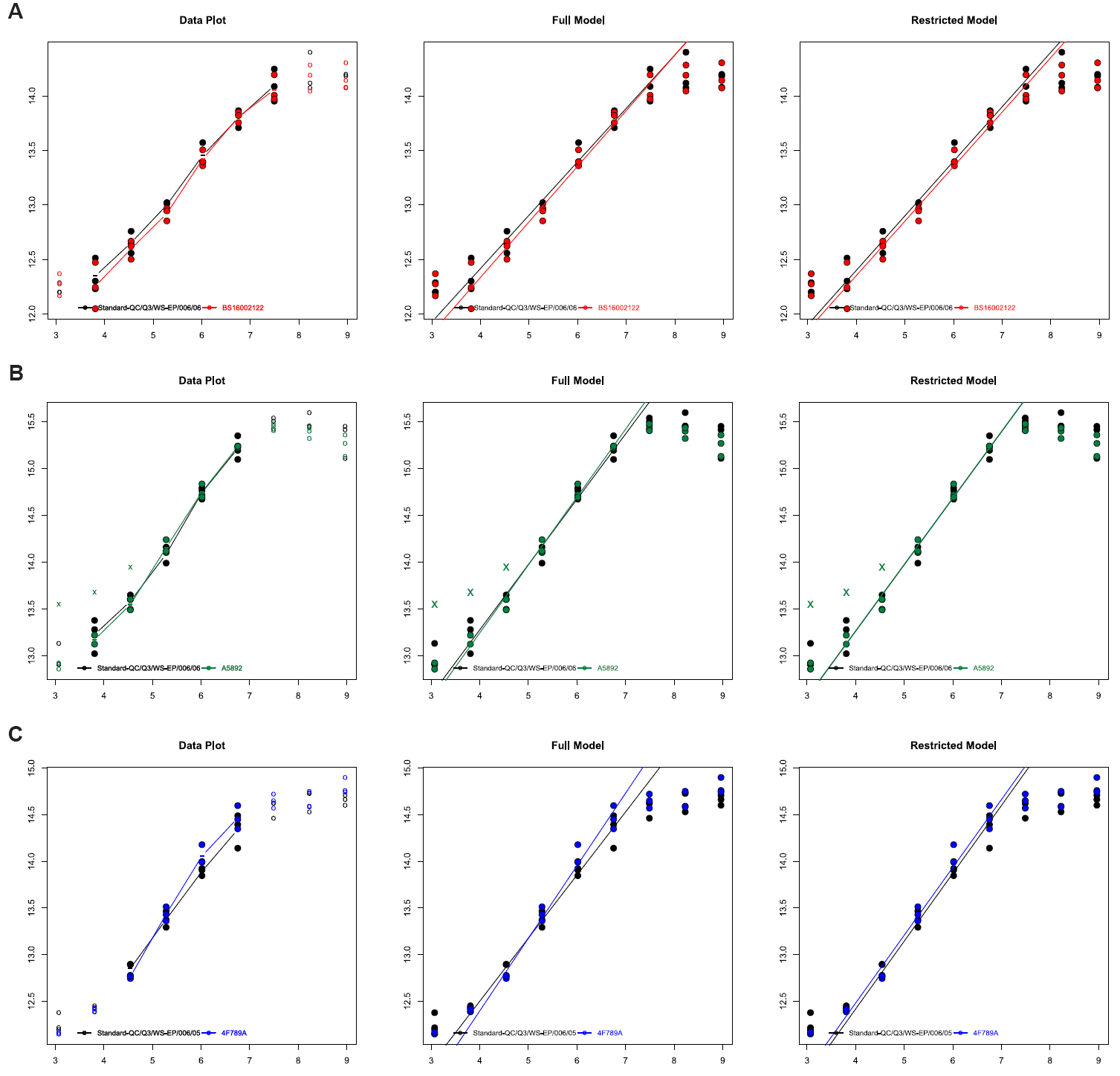

Supplement: S4 Fig — (A) MYL-1501D, (B) US-Licensed Insulin Glargine, and (C) EU-Licensed Insulin Glargine. (TIF) [file pone.0253168.s004.tif]

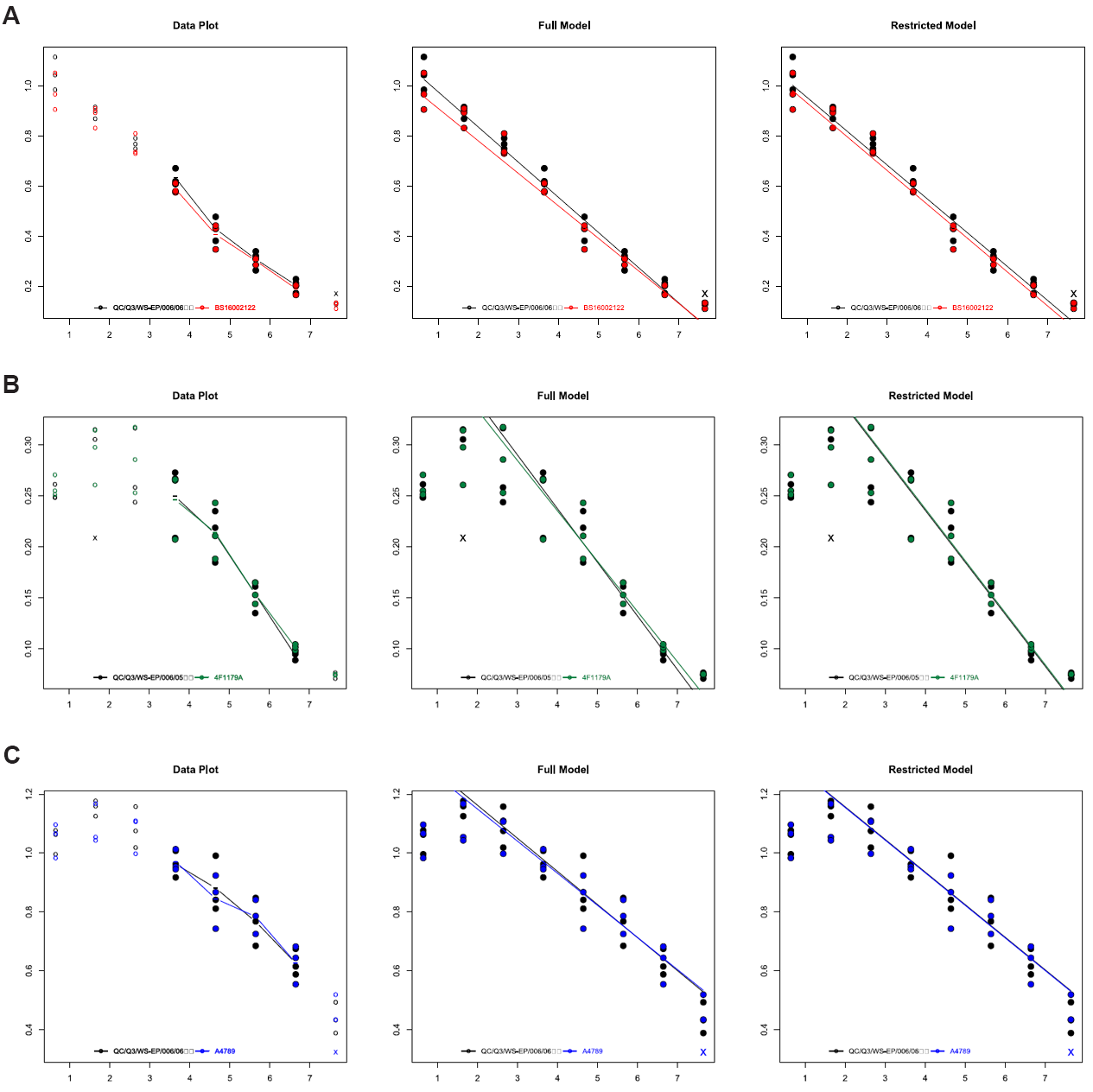

Supplement: S5 Fig — (A) MYL-1501D, (B) US-Licensed Insulin Glargine, and (C) EU-Licensed Insulin Glargine. PLA, parallel-line assessment. (TIF) [file pone.0253168.s005.tif]

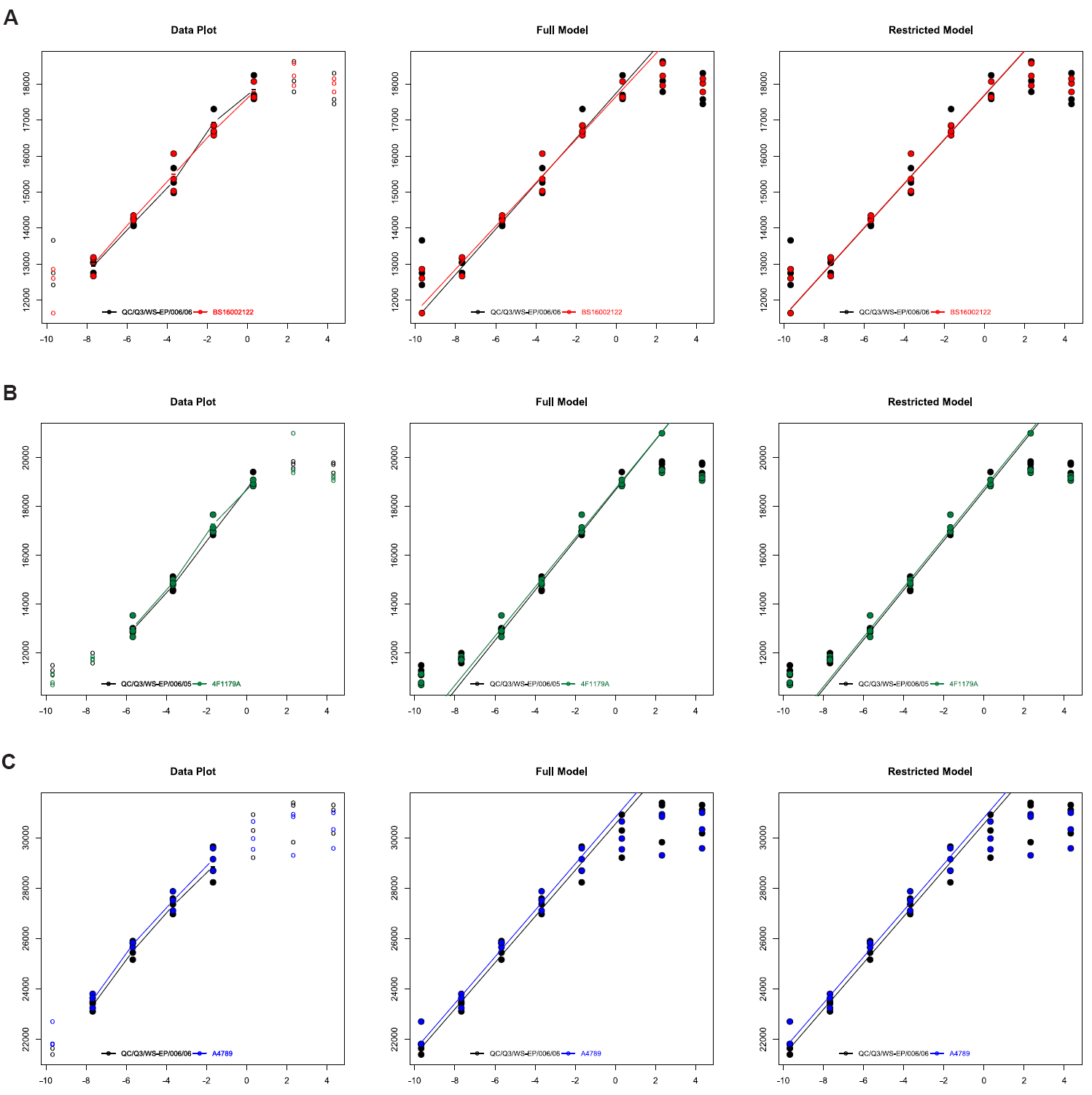

Supplement: S6 Fig — (A) MYL-1501D, (B) US-Licensed Insulin Glargine, and (C) EU-Licensed Insulin Glargine. (TIF) [file pone.0253168.s006.tif]
