# Supplementary material for: Physicochemical and functional characterization of MYL-1501D, a proposed biosimilar to insulin glargine
Source: PLoS One. 2021 Jun 16;16(6):e0253168. doi: 10.1371/journal.pone.0253168 (PMC8208551; doi:10.1371/journal.pone.0253168)
Supplement: S1 Table — (PDF) [file pone.0253168.s007.pdf]

S1 Table. Risk ranking of insulin glargine quality attributes

Very high	High	Moderate	Low
• Protein content<br>• Amino acid sequence<br>• Secondary structure<br>• Higher-order structure<br>• Functional activity<br>– Metabolic activity<br>– Mitogenic activity<br>– In vivo potency in rabbits	• Size variants (aggregates)	• pI variants (Des-TRR and Des-R)<br>• Conjugate variants (glycerol ester, citric acid conjugates, acetylation)	• Deamidation (B3 and A15)

Des-R, truncated protein missing 1 arginine amino acid from 1 end; Des-TRR, truncated protein missing 1 threonine and 2 arginine amino acids from 1 end; pI, isoelectric point.
